# Sex-specific placental transcriptome alterations in late-onset preeclampsia reveal male-biased immune and metabolic dysregulation

**DOI:** 10.1186/s13293-025-00781-w

**Published:** 2025-12-24

**Authors:** Melanie D. Smith, Seema Plaisier, James Breen, K. Justinian Bogias, Tanja Jankovic-Karasoulos, Dylan McCullough, Dale McAninch, Anya L. Arthurs, Melissa A. Wilson, Katherine A. Pillman, Claire T. Roberts

**Affiliations:** 1 Flinders Health and Medical Research Institute, College of Medicine and Public Health, Flinders University, Adelaide, SA 5042 Australia; 2 Comparative Genomics and Reproductive Health Section, Center for Genomics and Data Science Research, National Human Genome Research Institute, National Institutes of Health, Bethesda, MD 20852 USA; 3 Black Ochre Data Labs (Indigenous Genomics), The Kids Research Institute Australia & Australian National University, Adelaide, SA Australia; 4 National Centre for Indigenous Genomics, Australian National University, Canberra, ACT Australia; 5 Adelaide Medical School, University of Adelaide, Adelaide, SA 5005 Australia; 6 School of Biomedicine, University of Adelaide, Adelaide, SA 5005 Australia; 7 Centre for Cancer Biology, an alliance of SA Pathology, University of South Australia, Adelaide, SA Australia

**Keywords:** Late-onset preeclampsia, Placenta, Pregnancy, Transcriptomics, Extra-cellular matrix, Cell type deconvolution

## Abstract

**Background:**

Preeclampsia is a hypertensive disorder of pregnancy with major maternal and fetal consequences. While the molecular basis of early-onset preeclampsia is well studied, the mechanisms underlying late-onset disease—and how they differ by fetal sex—remain poorly understood. Placental transcriptomic profiling at term can reveal persistent molecular alterations reflecting cumulative disease processes.

**Methods:**

We conducted a cross-sectional observational analysis of placental gene expression using RNA sequencing in a subset of 58 term placentas (21 male-bearing and 37 female-bearing pregnancies) drawn from two large prospective birth cohorts. Pregnancies were classified based on a clinical diagnosis of late-onset preeclampsia (diagnosed ≥ 20 weeks’ gestation according to ISSHP criteria) or as uncomplicated pregnancies. We then assessed for differential gene expression. Cell type proportions were estimated using CIBERSORTx from a placenta-specific reference single-cell dataset. Weighted gene co-expression network analysis identified modules of co-expressed genes associated with late-onset preeclampsia and fetal sex.

**Results:**

Differential gene expression analysis identified 150 genes with altered expression in male-bearing placentas from pregnancies with late-onset preeclampsia compared to those from uncomplicated pregnancies. No differentially expressed genes were identified in female-bearing placentas. Cell type deconvolution revealed increased abundance of CD14 + monocytes and CD8 + activated T cells (log odds of 1.42 and 1.44 respectively) and reduced fetal GZMK natural killer cells (log odds of 0.60) in male-bearing placentas from affected pregnancies. In female-bearing placentas, late-onset preeclampsia was associated with increased fetal nucleated red blood cells and maternal plasma cells (log odds of 1.33 and 1.40 respectively). Male-specific co-expression analysis identified gene modules enriched for biological processes including RNA processing, immune regulation, and metabolism.

**Conclusions:**

Placental transcription and cellular responses to late-onset preeclampsia differ by fetal sex. Evidence of altered immune cell composition and gene co-expression in male-bearing placentas suggests a sex-specific vulnerability. These findings highlight the importance of considering fetal sex in molecular investigation and clinical management of preeclampsia.

**Plain english summary:**

Preeclampsia is a common pregnancy complication marked by high blood pressure, but how it affects the placenta, especially in later pregnancy and depending on the baby’s sex, is not well understood. In this study, we analysed placental tissue from pregnancies with and without late-onset preeclampsia using RNA sequencing. By separating the data based on whether the neonate was male or female, we found striking differences in gene expression. Only placentas from male-bearing pregnancies showed significant changes in gene expression linked to preeclampsia. These changes involved genes related to immune response, metabolism and vascular function. We also used computational tools to estimate what types of cells were present in each placental sample. In male-bearing pregnancies affected by late-onset preeclampsia, there was a notable increase in certain immune cells, suggesting an altered immune response and increased inflammation. In contrast, female-bearing pregnancies affected by late-onset preeclampsia showed an increase in cell composition for two blood related cell types, but no significant gene expression differences. By grouping genes that worked together into networks, we identified several groups, especially in placentas from male-bearing pregnancies, that were strongly associated with biological processes known to be disrupted in preeclampsia, such as blood vessel formation, extracellular matrix remodelling, and hormone regulation. These findings emphasise the importance of considering fetal sex in pregnancy research and could help guide future sex-specific diagnostic or treatment strategies.

**Supplementary Information:**

The online version contains supplementary material available at 10.1186/s13293-025-00781-w.

## Highlights

Sex-specific response: Significant placental gene expression changes between preeclamptic and uncomplicated pregnancies were found only in male-bearing pregnancies.

Immune involvement: Increased abundance of CD14 + monocytes and CD8 + T cells in placenta from male-bearing pregnancies suggests a heightened immune response.

ECM and vascular remodelling: Key gene modules in male-bearing placentas were enriched for extracellular matrix organisation and vascular function.

Transcriptomic network disruption: Co-expression analysis revealed distinct gene modules associated with preeclampsia in male-bearing pregnancies, reflecting changes in metabolism and gene signaling.

Female resilience hypothesis: Despite altered cell composition, female-bearing placentas showed no significant gene expression changes, suggesting possible adaptive resilience.

**Supplementary Information:**

The online version contains supplementary material available at 10.1186/s13293-025-00781-w.

## Background

Preeclampsia is a hypertensive disorder of pregnancy with significant maternal and fetal health implications, presenting after 20 weeks of gestation with new-onset hypertension and signs of organ dysfunction [1–4]. This condition is commonly classified into early-onset (diagnosed before 34 weeks’ gestation) and late-onset (diagnosed at or after 34 weeks) subtypes, which differ markedly in clinical presentation and pathophysiology [5–8]. Early-onset preeclampsia is characterized by impaired trophoblast invasion and defective maternal spiral artery remodelling, which compromises placental blood flow and damages chorionic villi and syncytiotrophoblast. The resulting cellular debris entering maternal circulation contributes to systemic inflammatory responses [9, 10]. In contrast, late-onset preeclampsia was traditionally considered a maternal disorder arising from underlying physiological predisposition, associated with morphologically normal placentas and normal fetal growth [6]. However, emerging evidence indicates that placental dysfunction also contributes to late-onset preeclampsia through mechanisms including syncytiotrophoblast senescence, reduced uteroplacental perfusion, and physical constraints of term gestation [11]. These changes may reflect “restricted placental capacity,” where the placenta cannot meet increasing fetal demands in late pregnancy. While initiating events occur earlier in gestation, analysis of term placental tissue can reveal cumulative disease effects and identify persistently altered biological pathways, providing insight into the mechanisms and consequences of late-onset preeclampsia pathophysiology.

Fetal sex is increasingly recognised as a biological factor influencing placental function and pregnancy outcomes. Epidemiological studies indicate that male-bearing pregnancies are associated with a higher risk of term preeclampsia, whereas female-bearing pregnancies are more often affected by early-onset disease [12–14]. Although some studies have found no association between fetal sex and preeclampsia risk [15–18], large cohort analyses and meta-analyses support an elevated risk in male-bearing pregnancies, particularly non-Asian and African American populations [19, 20]. These sex-specific differences may reflect underlying variation in maternal physiological adaptation, placental development, and fetal responses. Mechanistic studies suggest that male-bearing placentas exhibit enhanced pro-inflammatory signalling [21], altered vascular reactivity [22] and distinct transcriptomic landscapes [12, 23]. Despite this, most transcriptomic studies of preeclampsia do not stratify analysis by fetal sex, limiting the ability to identify sex-specific molecular pathways relevant to disease development.

In this study we investigate whether the placental transcriptome in late-onset preeclampsia differs by fetal sex. By examining gene expression in placental tissue from pregnancies with and without late-onset preeclampsia, we aim to identify transcriptional signatures that are specific to male- and female-bearing pregnancies. While the initiating events of late-onset preeclampsia occur earlier in gestation, analysis of term placental tissue can reveal cumulative disease effects and identify persistently altered biological pathways, providing insight into the mechanisms and consequences of late-onset preeclampsia pathophysiology. This sex-stratified approach addresses a critical gap in the current understanding of placental biology and preeclampsia, where fetal sex is often overlooked despite its well-established influence on disease risk and placental function.

## Methods

### Study participants

Patient samples were taken from both the Adelaide arm of the **SC**reening f**O**r **P**regnancy Endpoints (SCOPE) study [24, 25], and from the Adelaide based **S**creening **T**ests to predict poor **O**utcomes of **P**regnancy (STOP) study [26]. SCOPE was a prospective, international, multicentre cohort study of healthy, nulliparous, singleton-bearing women which recruited in Adelaide between November 2004 and September 2008. STOP was a prospective cohort study of healthy, nulliparous, singleton-bearing women which recruited between 2015 and 2018. For both studies, recruitment occurred at the Lyell McEwin Hospital, Adelaide, South Australia. Women were excluded from participation if they had ≥ 3 miscarriages or ≥ 3 terminations of pregnancy, major fetal anomalies, pre-existing hypertension on medication, Type I or Type II diabetes mellitus, renal disease, systemic lupus erythematosus, antiphospholipid syndrome, known major uterine anomaly or previous cervical cone biopsy. Exclusion criteria were applied to ensure recruitment of a low-risk pregnancy population and to minimise confounding from pre-existing conditions associated with pregnancy complications. Fetal and maternal outcomes were obtained from clinical records [27].

### Sample collection

Post delivery, term placentas were collected from pregnancies classified as being either uncomplicated, using the criteria described in [28] or as preeclamptic, using criteria described in [1, 29]. Placentas were collected immediately following delivery, after assessment by the midwife, to ensure tissue integrity and placed on a tray with the chorionic plate facing up and the basal plate in contact with the ice tray. To standardise sampling, the outer 2 cm perimeter was avoided, and tissue was dissected in a 2 cm-wide column extending inward from the edge. A 1 cm full-thickness block was taken at least 2 cm from both the placental edge and the cord insertion site and four 0.5 cm³ pieces were dissected from the middle. Samples were stored in RNAlater^®^ (Thermo Fisher) at 4 °C for 24 h before long-term storage at − 80 °C.

### Statistical analysis of clinical variables

Comparisons of clinical variables between preeclampsia and control groups were stratified by fetal sex (i.e., male preeclampsia vs. male control and female preeclampsia vs. female control). For normally distributed variables, maternal age, maternal BMI, and birthweight centile, group differences were assessed using unpaired two-sample Student’s *t*-tests. For variables that did not meet the assumption of normality, gestational age and birthweight, comparisons were performed using the non-parametric Wilcoxon rank-sum test (Mann-Whitney *U* test). All tests were two-sided and conducted in R (v4.05).

### RNA extraction and library Preparation

RNA was extracted from stored placental samples using the QIAGEN RNeasy Plus Mini Kit (QIAGEN, Hilden, Germany) following the manufacturer’s protocols except for substituting the RW1 buffer for 70% ethanol for better retention of smaller RNA fragments. Sequencing libraries were prepared using Illumina TruSeq Stranded Total RNA Sample Preparation kits and all the ribosomal RNA was depleted using Ribo-Zero Gold. Samples were pooled and sequenced on a single flowcell on the Illumina Hi-Seq 2500 using a 126 bp paired-end protocol (Flinders University Genomics Facility, South Australia).

### Data pre-processing and alignment

Each sample was sequenced to an average of 30 million reads. Given the sex-specific nature of our analyses, all pre-processing steps were conducted in parallel for male and female samples and performed on the Flinders University high-performance computing (HPC) system Deep Thought [30]. Raw sequencing reads in FASTQ format were assessed for quality control metrics using FASTQC (v0.12). Trimming of low-quality bases and adapters was performed using fastp (v0.23.2) with the parameters--cut_right, --cut_window_size 4, --cut_mean_quality 20, and --length_required 75. Post-trimming FASTQC quality metrics were aggregated using MultiQC (v1.14). Trimmed paired-end reads were aligned to the human genome (GRCh38) using STAR (v2.7.10a) with a sex-informed genome annotation from the XYalign package [31, 32]. This sex-chromosome aware alignment approach retains X and Y chromosome genes, masking the pseudo autosomal regions. This improves RNA quantification accuracy on the sex chromosomes and increases statistical power to detect sex-biased gene expression patterns [31]. Using the pre-built genome index, alignment was performed with the following parameters: --outFilterType BySJout, --outFilterMismatchNmax 999, --outSAMtype BAM SortedByCoordinate and --outSAMmapqUnique 60. Read group information was specified with --outSAMattrRGline.

### Duplicate removal and quantification

Read alignments were processed to remove PCR duplicates using Picard MarkDuplicates (v2.26.0, with -REMOVE_DUPLICATES=TRUE). Further details regarding the rationale and methodology for deduplication are provided in Supplementary Note 1. Gene-level quantification was performed using featureCounts (v2.0.3) with the following parameters: -a gencode.v29.annotation.gtf, -s 2, -p, --countReadPairs, and -T 16. The final matrix of gene counts aggregated across all samples (comprising 42,645 genes) was filtered using *edgeR* (v.3.16.5) [33] to remove lowly expressed genes (< 2 counts per million in ≥ 5 samples), resulting in retention of 15,030 genes for downstream analyses. *edgeR* was also used to normalise for library composition bias, distortions in expression comparisons caused by uneven RNA content between samples, using the Trimmed Mean of M values (TMM) as described in Robinson and Oshlack (2010) [33].

### Differential expression analysis

Differential expression analyses were conducted in the R statistical environment (v.4.0.5), using the *edgeR* (v.3.16.5) [33] and *limma* (v.3.30.11) [34] packages. *Limma-voom* was chosen given its performance with 6 or fewer samples per group [35]. Prior to differential expression analyses, the voom function from the *limma* package was used to model the mean-variance relationship in the data and compute sample-specific weights. These weights were then applied to the TMM-normalised log-counts to prepare the data for linear modelling. Differential expression analyses, including moderated F-statistic evaluation, adjusted p-value estimation, and log_2_ fold change analysis were performed using a moderated t-test with Benjamini-Hochberg (BH) multiple hypothesis test corrections as implemented in *limma*. A post-hoc power analysis (detailed in Supplementary Note 2) confirmed sufficient statistical power (>80%) to detect moderate-to-large expression changes (≥ 1.5-fold) in the male comparison, whereas reduced power for smaller effect sizes may have limited detection of more subtle transcriptional differences in female placentas.

The primary analysis employed a simple regression model without covariates for differential expression analysis; the justification for this approach and results from alternative models incorporating various covariates are detailed in Supplementary Note 2. Potential confounders including maternal age, body mass index (BMI), smoking status, and birthweight were evaluated for their impact on differential expression outcomes (see Supplementary Note 2). Adjusted models demonstrated high concordance with the outcome-only model, indicating minimal confounding. Therefore, the simple regression model was retained for the primary analysis to maximise interpretability and statistical power. After adjustment, gene expression was considered significantly different at false discovery rate (FDR) ≤ 0.05.

Sex-specific differential expression was assessed through post-hoc comparisons between four groups: control male, control female, preeclampsia male, and preeclampsia female, where ‘control’ refers to uncomplicated pregnancies and ‘preeclampsia’ refers to pregnancies complicated by late-onset preeclampsia. These comparisons controlled for other experimental factors to identify genes differentially expressed between sex-specific disease states.

### Cell type Deconvolution

To assess cell type proportions cell type deconvolution was performed using CIBERSORTx (v1.0) via a Docker container executed with Singularity. A publicly available single-cell reference matrix was used, originally generated from single-cell RNA-sequencing of 40,494 cells derived from placental chorionic villous samples [36]. Cell types common to maternal and fetal compartments were merged if they were predicted at extremely low abundance in the initial unmerged CIBERSORTx run (suggesting limited biological significance or detection sensitivity) or if they exhibited highly similar gene expression profiles, as shown by hierarchical clustering in the signature matrix heatmap (Supplementary Fig. 1). This methodological refinement addresses the inherent limitation of bulk RNAseq in distinguishing maternal from fetal contributions without SNP data, while enhancing the statistical power and interpretability of the deconvolution results. The merged cell types included immune components (B cells, CD14 + monocytes, NK/T cells, and various T cell subsets) and trophoblast populations, as detailed in Supplementary Table 1. Bulk RNA-seq data were deconvoluted in single-cell mode (--single_cell TRUE) with batch correction (--rmbatchSmode TRUE) and 100 permutations to output cell type fractions per sample.

### Correlation between gene expression and predicted cell type proportions

To explore associations between gene expression and predicted cell type composition, Pearson’s correlation coefficients were calculated between transcript per million (TPM) expression values and CIBERSORTx-derived cell type proportions. The analysis was restricted to genes identified as significantly differentially expressed in male placental samples. Correlations were performed separately for male and female samples to assess sex-specific patterns. For each gene, expression values were correlated per sample against the proportions of each predicted cell type.

### Statistical analysis of cell type abundance differences using beta regression

To assess differences in estimated cell type proportions between preeclampsia cases and uncomplicated controls, beta regression models were fitted separately for male- and female-bearing pregnancies using the *betareg* package in R, following the approach described in Campbell et al. [36]. Prior to modelling, cell type proportions were transformed using a continuity correction to constrain values strictly between 0 and 1. For each sex-stratified group, models were fitted for each cell type using the formula *proportion ~ Outcome + Gestational Age*, where *Outcome* indicates case or control status. Cell types with zero predicted abundance across all samples within a group were excluded from analysis. Models that failed to converge were also omitted. Odds ratios, 95% confidence intervals, and two-tailed Wald test *p*-values were extracted for the association with preeclampsia. A cell type was considered significantly different between groups if the 95% confidence interval did not include 1.

### Weighted gene co-expression network analysis

To identify co-expression modules within the DE genes (at 80% FDR confidence) and test the module association with cell type proportion, outcome and metadata characteristics of the pregnancy, a weighted gene correlation network analysis (WGCNA) was carried out using the WGCNA R package [58]. WGCNA was performed exclusively on male placental samples due to the absence of statistically significant differentially expressed genes in female samples. The rationale for sex-specific analysis is based on established biological differences in placental function and pregnancy outcomes between male- and female-bearing pregnancies [19]. To minimise confounding effects of sex-related variability on co-expression patterns, we evaluated metrics of sample variability, including the biological coefficient of variation (BCV) and gene-wise dispersion, which assess differences in expression heterogeneity across samples. These measures ensure that network detection reflects biologically meaningful co-expression rather than technical or sample-derived variability. Based on these assessments, co-expression network analysis was restricted to male-bearing pregnancies, allowing the identification of robust modules of co-expressed genes relevant to late-onset preeclampsia.

To focus the network analysis on genes with meaningful differential expression while retaining sufficient gene representation for module detection, we included only those genes with an adjusted *p*-value < 0.2 from the initial differential expression analysis. This threshold allowed us to enrich for genes with biological relevance without being overly stringent. Given that WGCNA applies a z-score transformation across samples, including genes with low variance or weak signal-to-noise ratios can artificially amplify noise and distort correlation structures. By filtering for moderately to highly responsive genes, we aimed to reduce this risk and enhance the biological interpretability of the resulting co-expression modules. The distance matrix was created using distOptions= “method=‘euclidean’”. The data was raised to a soft power of 24 and used to produce a tree from the topological dissimilarity matrix using method=“average”. To select gene modules, we performed dynamic branch cutting (method=“hybrid”, minClusterSize = 30, deepSplitParam = 4) and merged closely related modules using the mergeClose function (cutHeight = 0.15). Module eigengenes were determined and correlated with traits and the statistical significance of this was evaluated using the cor (use=“p”) and the corPvalueStudent functions. Module membership, a generalised version of intramodular connectivity, was calculated for each gene and module eigengene combination, defined as the correlation between the two. The resulting network was exported using the exportNetworktoCytoscape function (with weighted = TRUE, threshold = 0.05) visualised in Gephi [37].

### Gene set enrichment analyses

Gene set enrichment analysis (GSEA) was performed on ranked gene lists from differential expression results using GSEA software [38] using default parameters. Analysis was conducted against Molecular Signature Database (MSigDB v6.2) Hallmark gene sets (*n* = 50) and Immunologic signatures (C7 collection). Gene Ontology (GO) enrichment analysis of WGCNA modules was performed using the clusterProfiler package [39] with the enrichGO function. Significant enrichment was defined as Biological Processes terms with q-value < 0.05 after Benjamini-Hochberg multiple testing correction. To reduce redundancy among significant GO terms, results were simplified using the simplifyEnrichment package [40] for semantic clustering and the simplify function in clusterProfiler with a similarity cutoff of 0.7.

## Results

### Maternal participant information

The hospital from which the SCOPE and STOP participants were recruited serves a socioeconomically disadvantaged population. In both cohorts, nulliparous ostensibly low risk women with singleton pregnancies were recruited. Maternal characteristics are shown in Table 1.

**Table 1:** Demographic and clinical characteristics of 58 study participants stratified by sex and preeclampsia status

	Male		Female	
	Preeclamptic (*n* = 5)	Uncomplicated (*n* = 16)	Preeclamptic (*n* = 10)	Uncomplicated (*n* = 27)
Maternal age	23.4 (3.0)	24.2 (3.5)	25.7 (6.6)	25.1 (4.0)
First trimester BMI	33.5 (10.4)	26.8 (6.2)	31.7 (8.0)	25.6 (5.2)
Gestational Age (weeks)	38.7 (1.8)	40.2 (1.1)	38.7 (1.8)	40.2 (0.9)
Birthweight (g)	3411.4 (604.4)	3690.2 (364.6)	3095.3 (621.9)	3573.5 (377.0)
Birthweight Centile	42.4 (36.0)	56.5 (26.0)	43.1 (34.0)	56.9 (25.4)

Data is represented as mean (SD)BMI = maternal Body Mass Index

### Sex-specific gene-level differential expression analyses

**Fig. 1:**
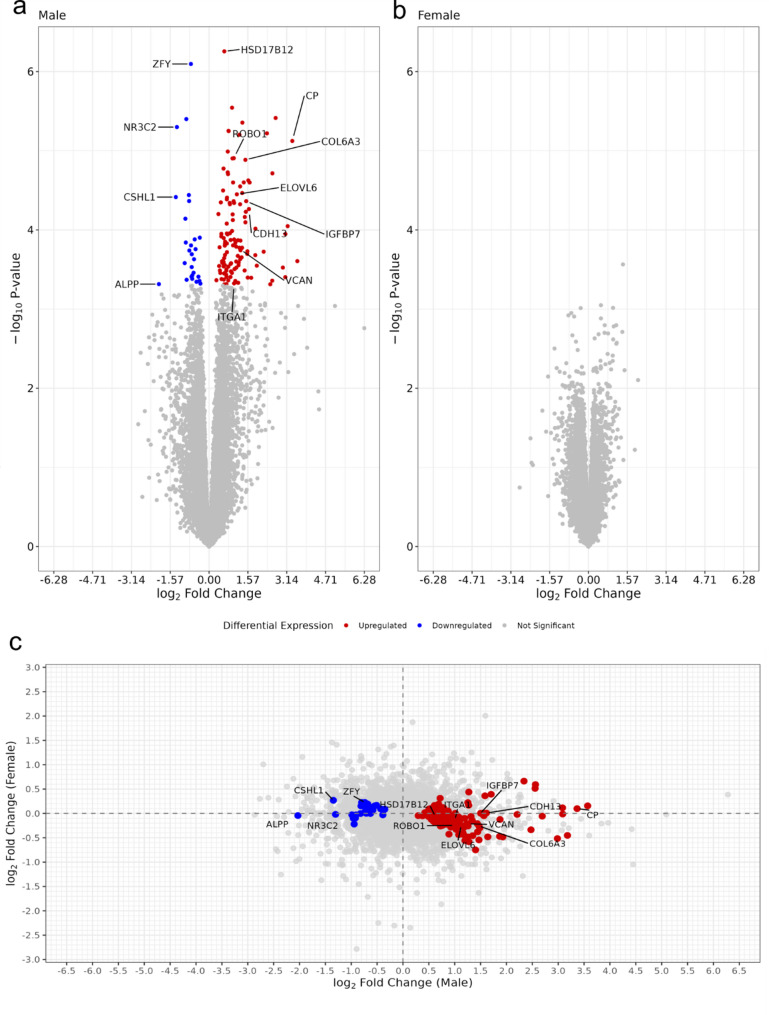
Differentially expressed genes (FDR < 0.05); indicated as red (up-) and blue (down-regulated in preeclampsia) in comparison of placental gene expression from preeclamptic and uncomplicated, male and female-bearing pregnancies. The volcano plots indicate the level of change (log_2_ fold) for male (**a**) and female (**b**) comparisons. The scatter plot (**c**) compares log_2_ fold change in gene expression between preeclamptic and uncomplicated pregnancies for male (x-axis) and female (y-axis) samples, with points coloured according to the results from differential expression in males (red points: upregulation in males – these display a variable female response; blue points: downregulation in males – these display minimal female response). No genes reached significance after multiple testing correction in the female group; unadjusted p-values are shown to enable visual comparison of nominal significance distributions between sexes

**Fig. 2:**
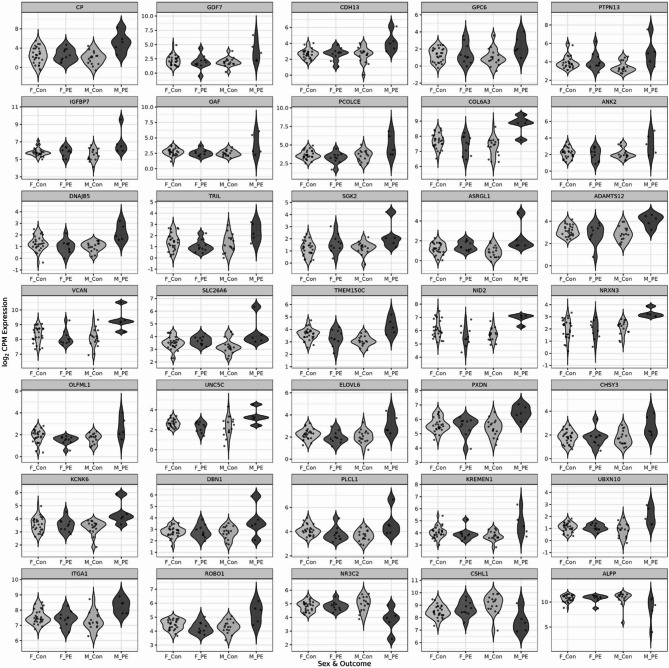
Expression of differentially expressed genes by fetal sex and outcome: Violin plots displaying the distribution of gene expression levels for the 35 most differentially expressed genes identified in the male analysis across the four outcome groups: Female Control (F_Con, light green), Female PE (F_PE, dark green), Male Control (M_Con, light purple), Male PE (M_PE, dark purple). Each panel represents a single gene (symbol in top panel), with normalised log_2_ gene expression values on the y-axis, with individual data points shown as dots. The consistent pattern across genes demonstrates Male PE samples exhibit distinct expression profiles whereas the other three groups display more similar expression patterns to one another

Linear modelling was performed to analyse and compare differential gene expression in late-onset preeclampsia and uncomplicated pregnancies stratified by fetal sex. From a total of 15,030 genes retained after filtering, we identified 150 genes that were differentially expressed (FDR < 0.05) in male-bearing pregnancies complicated by late-onset preeclampsia samples compared to uncomplicated male-bearing pregnancies (Table 2, Supplementary Table 2, Fig. 1a). In contrast, no genes met the significance threshold (FDR < 0.05) in the equivalent comparison of female-bearing pregnancies (Fig. 1b, Supplementary Table 3). Among the most notable findings in the male comparison was the differential expression of Ceruloplasmin (*CP*), encoding a copper-carrying protein crucial for iron metabolism and oxidative stress response, which has been previously implicated in hypertensive disorders of pregnancy [41] and in severity of preeclampsia [42]. Other significantly differentially expressed genes included Cadherin 13 (*CDH13*) and Insulin Like Growth Factor Binding Protein 7 (*IGFBP7*), both involved in vascular remodelling and angiogenesis [43, 44], as well as extracellular matrix components Collagen Type VI Alpha 3 Chain (*COL6A3*), integral in collagen organising matrix components and associated with hypertension [45] and Versican (*VCAN*), which plays critical roles in placental development including trophoblast migration and invasion [46]. The analysis also identified differential expression of genes involved in placental function and metabolism, including placenta-specific genes Chorionic Somatomammotropin Hormone Like 1 (*CSHL1*) and Placental Alkaline Phosphatase (*ALPP*), the mineralocorticoid receptor Nuclear Receptor Subfamily 3 Group C Member 2 (*NR3C2*), and ELOVL Fatty Acid Elongase 6 (*ELOVL6*). Additionally, Integrin Subunit Alpha 1 (*ITGA1*) and Roundabout Guidance Receptor 1 (*ROBO1*), which are important for cell-matrix interactions and angiogenesis respectively, showed significant differential expression. Visualising the log_2_FC from the sex-stratified comparisons reveals a sex-specific pattern of placental gene expression associated with late-onset preeclampsia, with many genes exhibiting not only male-specific significance but also a subtle yet consistent opposite direction of expression changes in female-bearing pregnancy compared to their male counterpart (Fig. 1c). Moreover, expression profiles of the 35 most differentially expressed genes reveal a clear pattern where female control, female preeclamptic and male control groups show greater similarity to each other than to the male preeclamptic samples (Fig. 2). The visualisation further highlights the divergence in expression patterns between sexes, with male preeclamptic samples showing pronounced differences in gene expression magnitude.

**Table 2:** Differentially expressed genes in placentas from male-bearing pregnancies with preeclampsia compared to controls

Ensembl ID	Gene name	logFC	Average log_2_ CPM expression				FDR (Males)
			Male control	Male PE	Female control	Female PE	
ENSG00000047457	*CP*	3.37	1.94	5.23	2.46	2.57	0.009
ENSG00000143869	*GDF7*	1.87	1.94	3.85	2.20	1.90	0.036
ENSG00000140945	*CDH13*	1.61	2.57	4.15	2.64	2.67	0.020
ENSG00000183098	*GPC6*	1.56	1.01	2.38	1.28	1.44	0.046
ENSG00000163629	*PTPN13*	1.55	3.44	5.01	3.95	4.05	0.034
ENSG00000163453	*IGFBP7*	1.50	5.50	6.95	5.82	5.84	0.018
ENSG00000184232	*OAF*	1.49	2.50	3.88	2.72	2.53	0.021
ENSG00000106333	*PCOLCE*	1.47	3.56	4.96	3.66	3.26	0.025
ENSG00000163359	*COL6A3*	1.47	7.32	8.80	7.67	7.40	0.012
ENSG00000145362	*ANK2*	1.44	2.10	3.28	2.19	1.99	0.023
ENSG00000137094	*DNAJB5*	1.35	0.99	2.21	1.30	0.98	0.009
ENSG00000255690	*TRIL*	1.27	1.29	2.30	1.42	1.04	0.037
ENSG00000101049	*SGK2*	1.27	1.25	2.26	1.19	1.61	0.034
ENSG00000162174	*ASRGL1*	1.27	0.91	2.31	1.21	1.47	0.032
ENSG00000151388	*ADAMTS12*	1.26	2.88	4.06	3.16	2.85	0.017
ENSG00000038427	*VCAN*	1.26	8.08	9.33	8.32	8.10	0.033
ENSG00000225697	*SLC26A6*	1.25	3.22	4.33	3.46	3.67	0.018
ENSG00000249242	*TMEM150C*	1.23	3.07	4.26	3.59	3.32	0.009
ENSG00000087303	*NID2*	1.20	5.71	6.93	6.11	5.57	0.038
ENSG00000021645	*NRXN3*	1.19	2.13	3.28	2.04	1.93	0.040
ENSG00000183801	*OLFML1*	1.17	1.65	2.63	1.90	1.50	0.032
ENSG00000182168	*UNC5C*	1.16	2.36	3.34	2.64	2.20	0.036
ENSG00000170522	*ELOVL6*	1.12	2.06	3.14	2.26	1.97	0.018
ENSG00000130508	*PXDN*	1.10	5.41	6.49	5.64	5.50	0.038
ENSG00000198108	*CHSY3*	1.10	1.75	2.78	1.81	1.74	0.040
ENSG00000099337	*KCNK6*	1.10	3.34	4.40	3.55	3.52	0.033
ENSG00000113758	*DBN1*	1.06	2.72	3.71	2.81	2.80	0.047
ENSG00000115896	*PLCL1*	1.03	3.66	4.69	4.09	3.79	0.041
ENSG00000183762	*KREMEN1*	1.01	3.77	4.76	4.04	3.90	0.032
ENSG00000162543	*UBXN10*	1.01	0.88	2.01	1.10	1.07	0.018
ENSG00000213949	*ITGA1*	1.01	7.27	8.28	7.55	7.42	0.049
ENSG00000169855	*ROBO1*	1.00	4.30	5.28	4.46	4.23	0.012

Ensembl ID	Gene name	logFC	Average log_2_ CPM expression				FDR (Males)
			Male control	Male PE	Female control	Female PE	
ENSG00000151623	NR3C2*	−1.30	4.98	3.81	4.84	4.84	0.009
ENSG00000204414	*CSHL1*	−1.35	9.08	7.70	8.49	8.71	0.018
ENSG00000163283	*ALPP*	−2.03	10.77	8.58	10.75	10.72	0.049

Differential expression analysis identified 123 upregulated and 27 downregulated genes (FDR < 0.05) in male preeclamptic placentas compared to male controls. This table presents top genes with |logFC| ≥ 1 and average log_2_ CPM ≥ 1. Columns show average log_2_ CPM for the male comparison. Genes are ordered by decreasing logFC. * Transcription Factor

### Functional enrichment analysis of differentially expressed genes

To investigate potential function coordination within the differentially expressed genes in male late-onset preeclampsia, we applied GSEA [38, 47] to test for enrichment of curated gene sets, groups of genes that share a common biological function, chromosomal location or regulation. Enrichment in a given gene set can be identified where the members of the DE table, rather than be randomly distributed, are primarily found at the top (up-regulated in a gene set) or the bottom (down-regulated in a gene set) of a curated set [38]. Rather than focus on individual genes, GSEA identifies enrichment of biological pathways or processes. We identified enrichment for 14 GO Biological Pathway terms with an FDR q-value < 0.05 (Supplementary Table 4). Notably, processes related to RNA processing and transport were significantly enriched, including RNA and mRNA export from the nucleus (NES = −2.38, FDR q-val = 0.0 and NES = −2.23, FDR q-val = 0.002, respectively). Processes associated with mRNA transport (NES = −2.2, FDR q-val = 0.003) and vesicle targeting (NES = −2.13, FDR q-val = 0.006) were also significantly enriched, underscoring potential perturbations in intracellular trafficking mechanisms crucial for proper mRNA localisation and protein synthesis. Similarly, enrichment of RNA splicing via transesterification reactions (NES = −2.07, FDR q-val = 0.011) and mRNA processing (NES = −2.07, FDR q-val = 0.014) highlights dysregulation in RNA maturation pathways, which are essential for generating functional transcripts. Interestingly, embryonic placenta morphogenesis (NES = −1.96, FDR q-val = 0.044) was also enriched, suggesting that transcriptional changes in genes associated with placental development and structure may contribute to the observed phenotype. Additionally, the significant enrichment of nucleobase biosynthetic processes (NES = 2.06, FDR q-val = 0.023) may indicate compensatory upregulation of nucleotide synthesis pathways to counterbalance deficits in RNA-related processes.

### Cell proportion Deconvolution of RNAseq from bulk placental tissue

We applied cell-type deconvolution using CIBERSORTx with the placenta specific signature matrix to estimate cell type proportions from bulk placental tissue in all samples. Among the signature genes, original bulk and estimated mixtures had a Pearson correlation (mean ± standard deviation) of 0.78 ± 0.07 and root mean square error of 0.72 ± 0.05 (Supplementary Table 5). The Pearson correlation of 0.78 ± 0.07 between original bulk and estimated mixtures indicates good deconvolution performance, consistent with benchmarks for reliable cell type proportion estimates [48].

First, we analysed variation in cell type proportion across all 58 placentas independent of pregnancy outcome or neonatal sex. The analysis revealed that villous cytotrophoblasts were the most abundant cell type, comprising a mean of 16.95% ± 4.33% of the total cell population for all samples. Fetal plasmacytoid dendritic cells (10.30% ± 3.33%) and naive CD8 + T cells (10.11% ± 3.06%) were the next most prevalent cell types. Fetal extravillous trophoblasts exhibited the highest relative variability, with a standard deviation of 5.42%, representing more than half their mean proportion (9.27%), indicative of substantial heterogeneity. Natural killer T cells displayed no variability, consistently measured at 0% and were removed from downstream analyses. Several cell types were present at very low abundances (< 1%), including maternal FCGR3A + monocytes (0.32% ± 0.89%), fetal memory CD4 + T cells (0.21% ± 0.58%), naive CD4 + T cells (0.08% ± 0.38%), and maternal plasma cells (0.09% ± 0.45%).

**Fig. 3:**
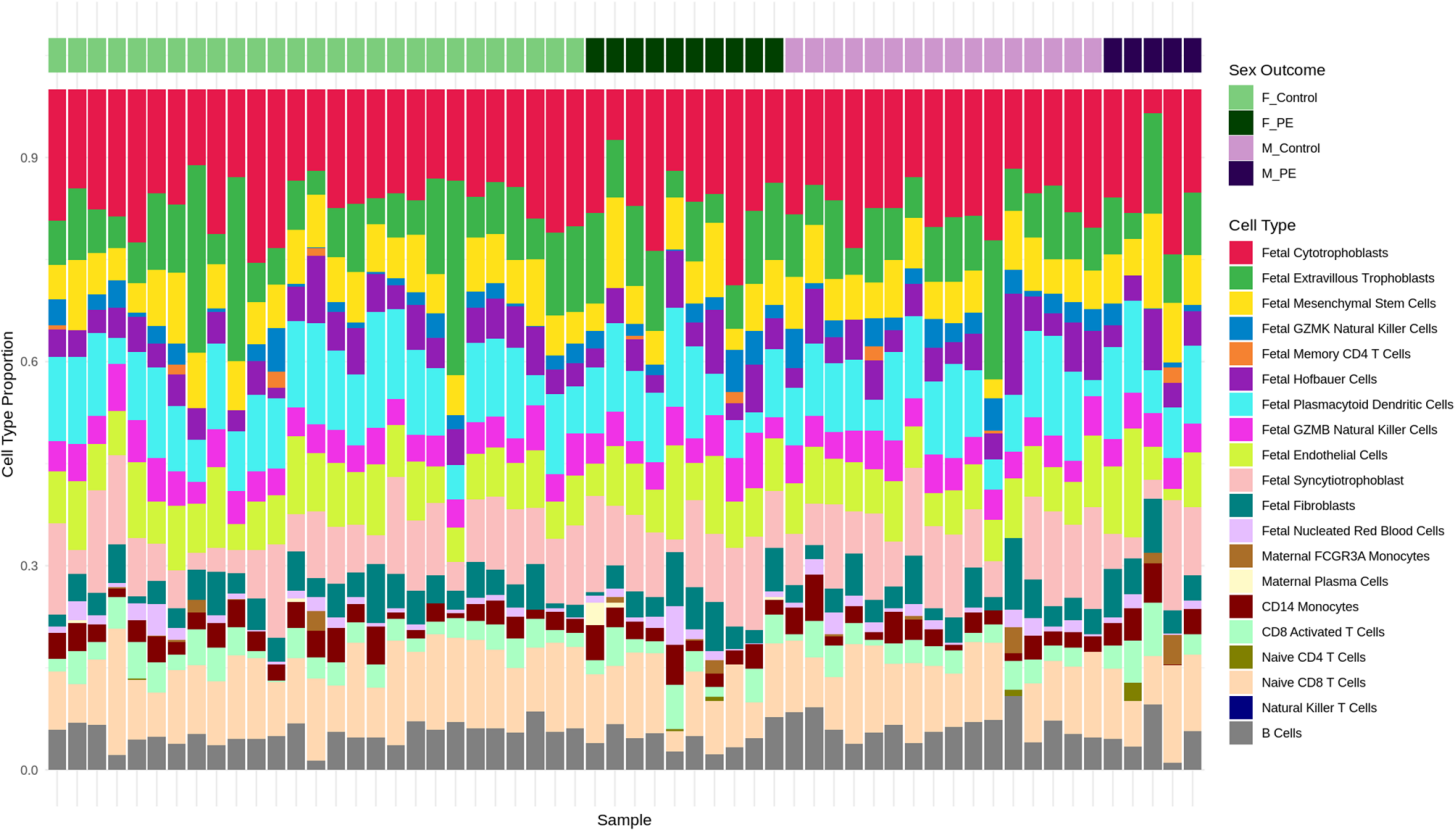
Predicted cell type proportions derived from placental gene expression data: Stacked bar chart showing the proportions of cell types in placental samples post-deduplication. Each bar represents an individual sample, with cell type proportions indicated by different colours. Samples are grouped by fetal sex and outcome (4 outcome groups: Female Control (F_Control, light green), Female PE (F_PE, dark green), Male Control (M_Control, light purple), Male PE (M_PE, dark purple)). Cell type annotations highlight the heterogeneity of cellular composition across samples, with notable variation observed between outcomes and sex

The composition profile (Fig. 3) reflects the expected diversity of a placental sample, with contributions from both fetal and maternal cell populations. Trophoblast-related cells, including cytotrophoblasts, syncytiotrophoblasts, and extravillous trophoblasts, were prominent, alongside a significant presence of immune cells, particularly of fetal origin. Overall, most cell types exhibited moderate variability, with standard deviations around 1–3% of their mean values. However, the higher variability observed in some cell types may suggest biological heterogeneity within the placental environment or technical variation in the deconvolution process.

### Differentially abundant cell type proportions in placentas from preeclamptic versus uncomplicated pregnancies

**Fig. 4:**
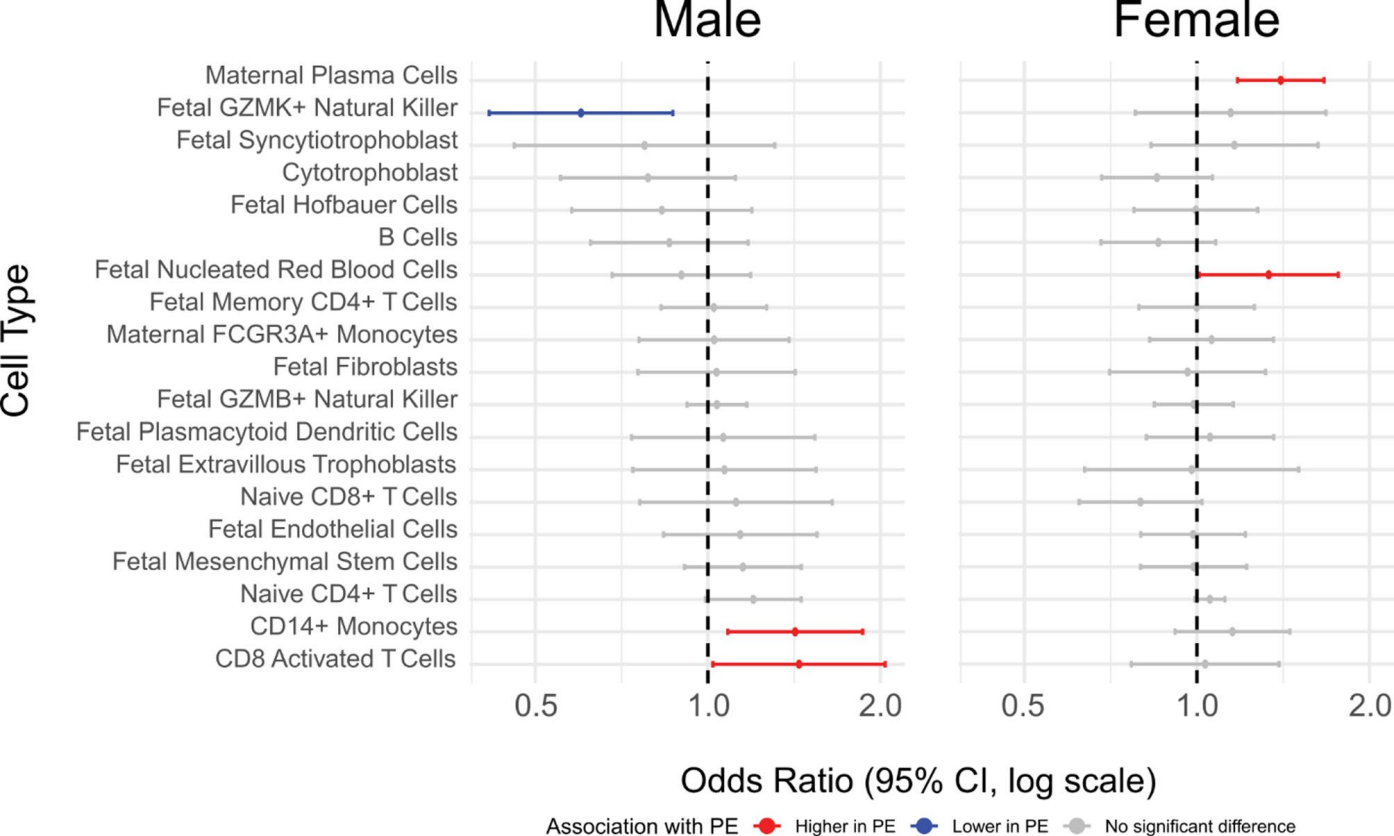
Sex-stratified cell type proportions in preeclampsia cases versus uncomplicated controls. Forest plots show prevalence odds ratios derived from multivariate beta regression models for male (left; *n* = 5 cases, 16 controls) and female (right; *n* = 10 cases, 27 controls) placental samples. Horizontal lines represent 95% confidence intervals. As maternal plasma cells were not predicted in any male samples, no horizontal line is displayed in this group

Our investigation into cellular heterogeneity revealed distinct sex-specific alterations in cell type proportions associated with preeclampsia. To systematically assess these differences in cell proportions between preeclampsia cases and controls, we fitted a sex-specific beta regression model for each cell type proportion to estimate the prevalence odds ratio for each cell type, representing the odds of higher or lower cell abundance in preeclamptic relative to uncomplicated pregnancies (Supplementary Tables 6 and 7; log odds ratio for male and female analyses). We found maternal plasma cells (p-value < 0.001) and fetal nucleated red blood cells (p-value = 0.04) in significantly higher proportions in placenta from preeclamptic female-bearing pregnancies (Fig. 4), while placentas from preeclamptic male-bearing pregnancies exhibited significantly fewer fetal GZMK-natural killer cells (p-value = 0.007) and higher proportions of CD14 + monocytes (p-value = 0.01) and CD8 + activated T cells (p-value = 0.04) (Fig. 4).

### Correlation between differential gene expression and cell type proportions

**Fig. 5:**
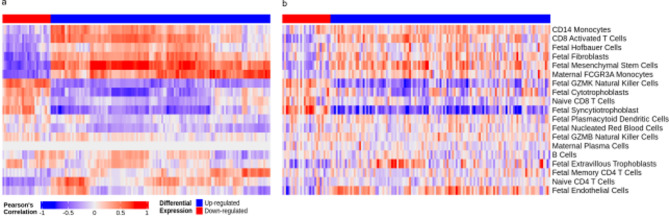
Heatmap of Pearson’s correlation coefficients between gene expression and predicted cell type proportion for genes differentially expressed in male samples. Gene expression values (transcripts per million) were correlated with cell type proportions predicted using CIBERSORTx. Colour intensity indicates the strength and direction of correlation. In male samples (**a**), distinct and often strong correlation patterns are observed, with hierarchical clustering on genes (columns) reflecting the direction of differential expression. In contrast, when female sample data are displayed (**b**) using the gene ordering derived from male clustering, markedly weaker correlations with much higher variation between genes are observed between gene expression and cell type proportions

To assess whether the expression of differentially expressed genes was associated with variation in cell type composition, we correlated gene expression with predicted cell type proportions (Fig. 5). The heatmaps demonstrate that various subsets of the differentially expressed genes, identified exclusively in the male comparison, exhibit distinct and sometimes strong correlation patterns with the presence of specific cell populations. In male preeclamptic placentas (left panel), hierarchical clustering identified clear sets of genes that strongly correlate with cell type fractions, suggesting these gene signatures may reflect, or respond to, underlying shifts in sample cellular composition. Consistent with the most significantly enriched cell-types identified in male preeclamptic placentas (Fig. 4), this analysis highlights gene clusters positively associated with CD14 + monocytes and CD8 + activated T cells. These associations align with the direction of cell-type enrichment observed in male placentas from late-onset preeclampsia (Fig. 4), where elevated odds ratio suggested increased proportion of these cell types, although the result did not reach statistical significance. Together these findings suggest that increased presence of, or transcriptional signaling by or in response to, fetal mesenchymal cells may contribute to the altered placental immune-stromal environment associated with late-onset preeclampsia in male bearing pregnancies [49]. Conversely, a cluster of genes exhibit strong negative correlations with fetal GXMK + natural killer cells, also consistent with their reduced presence in male preeclampsia (Fig. 4). Interestingly, when the same genes (derived from male differentially expressed genes) were examined in female samples (right panel), and gene ordering from the male clustering was imposed enabling direct visual comparison, the correlation structure in females appeared markedly different, exhibiting generally weaker and substantially less consistent gene-level correlations.

### Constructing a co-expression network of the human placenta

Gene correlation networks provide a powerful framework to explore coordinated gene expression changes, offering insight into the differential activity of biological pathways. In our dataset, BCV and dispersion were comparable between male and female samples (BCV: 0.367 vs. 0.342; dispersion: 0.135 vs. 0.117), indicating that the absence of detectable differential expression in female-bearing placentas is unlikely to be due to increased variability or differences in data quality. Based on this, WGCNA was restricted to male samples, enabling identification of robust co-expression modules specific to male-bearing pregnancies. WGCNA leverages the coordinated behaviour of gene modules rather than relying on individual genes, making it robust to the inclusion of weakly differentially expressed genes and allowing aggregation across co-expressed groups to buffer against noise.

Weighted gene co-expression network analysis was performed on 2,077 genes with FDR < 0.2 in male samples. Network construction initially identified 14 co-expression modules, which were subsequently merged to yield 9 distinct modules labelled by colour (Supplementary Fig. 2). Module expression patterns were visualised across fetal sex and pregnancy outcome (Supplementary Fig. 3), revealing transcriptional signatures associated with preeclampsia in male-bearing placentas. As expected, genes in the grey module demonstrated poor co-expression and were excluded from downstream analyses. The remaining eight modules ranged in size from 526 genes (light blue) to 79 genes (green). For details see Table 3.

**Table 3:** Weighted gene Co-expression network analysis (WGCNA) modules associated with preeclampsia in male-bearing placentas

Module	No. of genes	No. of DE genes	Variance explained by eigengene	Top ten hub genes (kME)	Module gene ontology after semantic reduction	Adjusted *P*-Value
Light blue	526	17	0.57	*CDC42SE1*,* KAT5*,* RBM23^*,* DDX59*, ATF1*,* CNST*,* POR*,* RASA1*,* CDYL*, SP3*	Vesicle targeting regulation of protein catabolic process histone modification RNA splicing, via transesterification reactions with bulged adenosine as nucleophile glycolipid biosynthetic process regulation of protein complex stability	3.36E-04 3.36E-04 1.79E-03 2.15E-03 1.37E-02 2.21E-02
Orange	453	61	0.42	*NID1*,* VCAN*,* KCNK6*, MEIS1*,* FARP1*,* IGFBP7*,* EMILIN2*,* CCDC88A*,* BST1*,* BVES*	Extracellular matrix organization cell-substrate adhesion sensory system development striated muscle hypertrophy	8.32E-05 2.05E-04 2.05E-04 1.10E-02
Dark blue	329	10	0.37	*SECISBP2*,* DYRK1A*, RORA*,* USP7*,* CLIP1*,* KAT6A*,* NUP153*,* RABGAP1*,* PIP5K1A*,* CREBRF*	Histone modification	5.47E-03
Black	311	22	0.47	*KCTD20*,* IPO5*,* PPIA*, ZNF641*,* ADH5*,* S100A10*,* MPHOSPH6*,* ATAD2*,* HMGB1*,* SCLT1*	Small molecule catabolic process aminoacyl-t RNA metabolism involved in translational fidelity	3.55E-02 3.55E-02
Pink	139	15	0.56	*FAR1*,* R3HDM1*,* LRP12*,* ABCC5*,* RUFY3*,* MAP9*,* RIC8B*, ZNF431*,* NEK1*,* SDHC*	Regulation of integrin-mediated signaling pathway regulation of basement membrane organization	1.45E-02 2.92E-02
Red	119	17	0.25	*OMD*,* ECM2*,* ADCY1*,* PTPN13*,* LINC02600*,* AVPR1A*,* RXFP1*,* MGST1*,* GALNT13*,* IGFBP1*	Regulation of vasoconstriction	2.47E-02
Purple	96	3	0.53	*ASPM*,* LMNB1*,* TYMS*,* ALDH7A1*,* CENPE*,* FANCI*,* MTHFD1*,* CKAP2L*,* CKAP2*,* CENPF*	Chromosome segregation	1.1E-07
Green	79	5	0.31	*ITGA9*,* HIST1H4I*,* RACGAP1*,* GPRC5C*,* CISD1*,* CYSTM1*,* TNFRSF1B*,* HIST1H3A*,* HIST1H3C*,* HIST1H2BL*	Isoprenoid biosynthetic process	4.22E-03

This table summarises the key characteristics of gene co-expression modules identified through WGCNA analysis of differentially expressed genes in male-bearing placentas from preeclamptic and uncomplicated pregnancies. For each module, the number of genes, number of differentially expressed genes (adjusted p-value < 0.05), variance explained by the module eigengene, top ten hub genes ranked by module eigengene-based connectivity (kME), and significantly enriched Gene Ontology (GO) terms after semantic reduction are shown. Modules are organised by size (number of genes), with adjusted p-values provided for each GO term. * Transcription factor ^ Splicing factor

### Functional analysis of placental gene co-expression modules

**Fig. 6:**
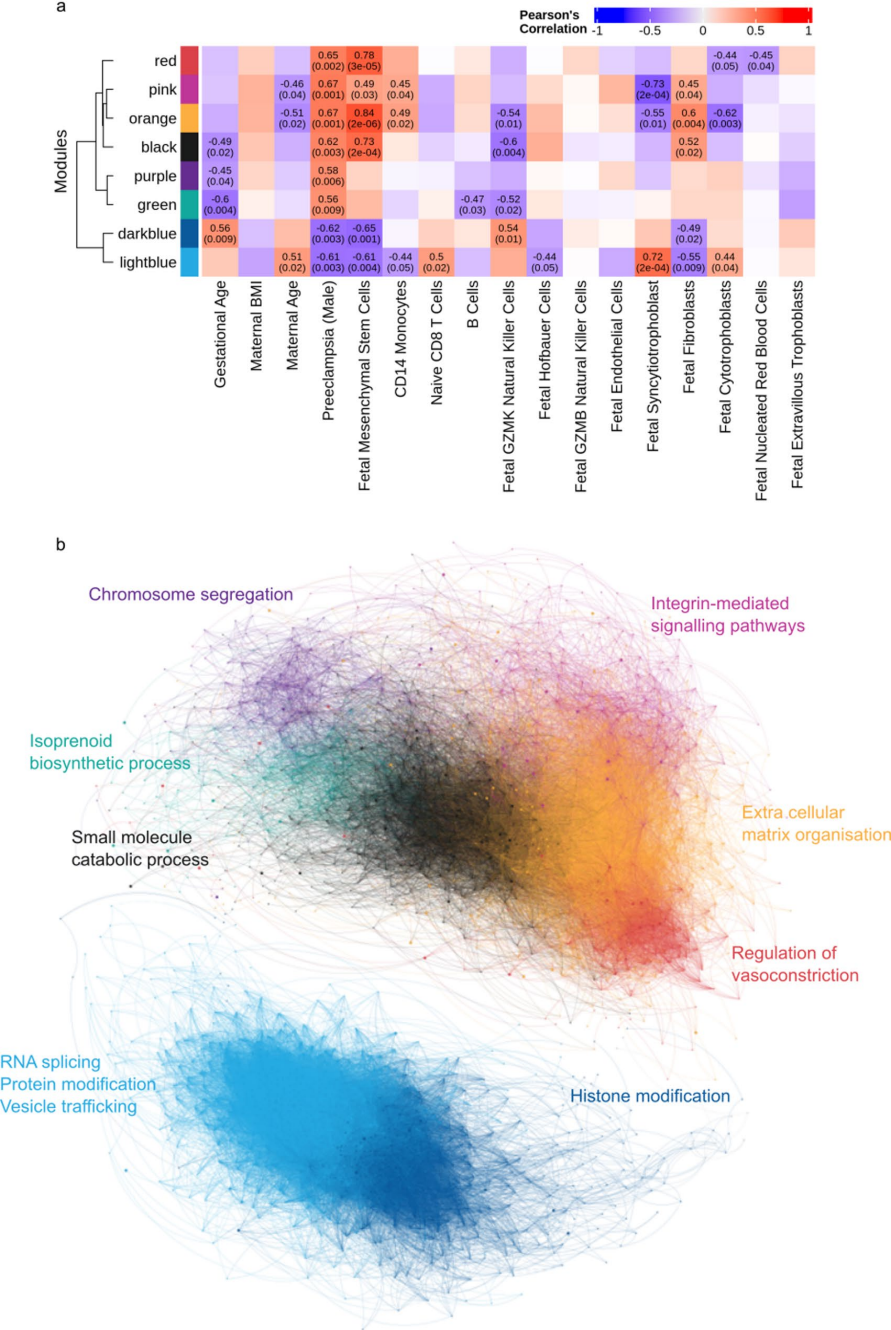
Module–trait relationships and co-expression gene networks. **(a)** Heatmap of correlations between module eigengenes (rows) and study traits or metadata (columns). Positive correlations are shown in red and negative correlations in blue, with colour intensity indicating the strength of the correlation. Each cell displays the correlation coefficient, with the corresponding *p*-value in parentheses. Modules such as red, orange, and black show strong positive correlations with the proportion of fetal mesenchymal stem cells, whereas the light blue module is strongly correlated with fetal syncytiotrophoblasts. **(b)** Gene co-expression networks derived from RNA-seq data from term placentas from uncomplicated (*n*= 16) and preeclamptic (*n* = 5) pregnancies, post-delivery. Nodes (circles) represent individual genes, and edges (lines) represent co-expression relationships. Networks were constructed using a signed adjacency matrix and clustered using hierarchical clustering with dynamic branch cutting, yielding nine distinct modules. Modules are clearly separated into down-regulated (light and dark blue) and up-regulated (all other) gene groups. For each module, the most significant Gene Ontology (GO) term after semantic reduction is indicated, with module colour corresponding to the heatmap in panel **(a)**

To shed further light on the basis of the differential transcriptome expression between placentas from preeclamptic and uncomplicated pregnancies, we both correlated module eigengenes against clinical variables and estimated cell type proportions (Fig. 6a) and performed gene ontology enrichment analysis (visualised in a graph representation of the correlation network; Fig. 6b). The graph structure revealed the modules to be highly intra-connected with the green and pink modules showing more inter-module connection than other modules. Gene ontology analysis (Supplementary Table 8) revealed distinct biological processes associated with specific WGCNA gene modules, several of which have clear potential to impact placental biology.

Six modules were positively correlated with late-onset preeclampsia outcome – the orange, red, black, pink, green and purple modules – consistent with a positive role in preeclampsia-related processes. Among these, the orange and red modules showed strong correlations with fetal mesenchymal stem cell proportions (see Fig. 6a), consistent with activation of these genes in such cells themselves or elsewhere in the placentas they are enriched in. The orange module was enriched for developmental processes and extracellular matrix organisation, including cell adhesion and tissue morphogenesis, functions characteristic of mesenchymal stem cells. The red module was specifically enriched for vasoconstriction and blood circulation regulation, processes notably dysregulated in preeclampsia. The black module also showed positive correlation with mesenchymal stem cells (see Fig. 6a) and was enriched for translational processes.

In contrast, the pink, purple and green modules all showed no strong positive correlation with any cell type, suggesting the observed gene expression differences are more likely due to changes within cell types rather than changes in cell type proportions. The pink module was enriched for integrin-mediated signalling pathways, which are help in help in cell attachment, cellular communication and migration [50] and by mediating cell adhesion and interactions between the conceptus and the uterine wall during implantation and placentation [51]. The purple module was enriched for cell cycle regulation and chromosome segregation, essential processes for placental development and plausibly linked to the aberrant placentation observed in preeclampsia. The green module was enriched for sterol metabolism, a process essential for placental hormone production.

Two modules were negatively correlated with preeclampsia outcome. The light blue module was strongly correlated with fetal syncytiotrophoblast (see Fig. 6a) and cytotrophoblast proportions (see Fig. 6a), as well as maternal age (see Fig. 6a). The light blue module was enriched for fundamental cellular processes including RNA splicing, protein modification, and vesicle trafficking - consistent with the high metabolic demands of syncytiotrophoblasts in healthy pregnancies. The dark blue module, positively correlated with gestational age (see Fig. 6a) and was enriched for histone and peptidyl-lysine modifications, suggesting reduced epigenetic activity in preeclampsia.

The eight modules identified ranged from 79 to 526 genes and exhibited distinct correlation patterns with clinical variables and cell type proportions, with six modules positively correlated and two negatively correlated with late-onset preeclampsia outcome. The dark blue and light blue modules appear linked to regulatory and metabolic activities in healthy placental function, respectively, while modules such as orange and red reflect altered extracellular matrix and vascular processes in preeclampsia. These results provide insights into the functional and cellular complexity of the placental transcriptome in health and disease.

## Discussion

In this study, we investigated the transcriptional landscape of late-onset preeclampsia through a sex-specific lens, uncovering distinct molecular signatures between male- and female-bearing placentas. Striking sex-dependent transcriptional differences were evident, with significant differential gene expression observed exclusively in placentas from male-bearing pregnancies. These findings highlight the importance of considering fetal sex in understanding preeclampsia pathophysiology, which may help explain inconsistencies reported in previous studies [52, 53]. By modelling both fetal sex and outcome in our regression and downstream analyses, we provide new insight into the molecular mechanisms underlying preeclampsia and underscore fetal sex as a key determinant in disease pathophysiology.

### Placental differential expression analysis

Our study investigated differentially expressed genes in placental tissue from male-bearing pregnancies affected by late-onset preeclampsia. While power analysis suggests that our study was adequately powered to detect moderate expression differences, the absence of significant DEGs in female-bearing placentas may also reflect biological or technical factors. It is possible that transcriptional responses to late-onset preeclampsia are more subtle or cell type–specific in female placentas and therefore less likely to be detected in bulk RNA-seq data. We found the male-specific transcriptional signature in late-onset preeclampsia was characterised by 150 differentially expressed genes, including several with established roles in placental function and vascular regulation. Comparison with network-based analyses of PE placentas [54] showed limited overlap with our male-specific DEGs, likely due to differences in sex stratification, disease subtype and analytical framework. While early-onset preeclampsia is often linked to severe placental vascular dysfunction, late-onset preeclampsia is more commonly associated with maternal systemic factors and less pronounced placental abnormalities [55]. However, our findings suggest that significant alterations in several biological pathways may exist in male-specific late-onset preeclampsia, potentially masked in previous studies by their sex-specific nature. Collectively, these findings suggest that male-bearing placentas exhibit a pattern of transcriptional changes indicative of reduced adaptive capacity to maternal or environmental stressors. Heightened inflammatory activation, altered vascular–stromal remodelling, and impaired metabolic signalling may act synergistically to compromise placental resilience, potentially explaining the greater susceptibility of male fetuses to adverse outcomes in preeclampsia. These mechanisms provide a unifying framework for understanding sex-specific vulnerability in late-onset disease. Here, we discuss the functional categories of the identified differentially expressed genes and their potential implications for placental function.

### Inflammatory and immune response

Higher expression of genes such as *TRIL*, *CP*, and *IGFBP7* suggest a heightened inflammatory response and oxidative stress. Late-onset preeclampsia is often accompanied by maternal systemic inflammation, which could influence placental gene expression. The upregulation of *TRIL* suggests a role for TLR4-mediated inflammatory pathways. *CP*, a copper-carrying protein involved in iron metabolism, serves a dual role - both as a protective mechanism against oxidative stress through its ferroxidase activity and as a marker of the inflammatory response characteristic of preeclampsia. This finding aligns with previous studies implicating oxidative stress in preeclampsia pathogenesis, with our data suggesting this effect may be more pronounced in male-bearing placentas. *IGFBP7*, a key regulator of cell growth, is associated with cellular senescence, tissue aging, and obesity, and its high concentrations have been linked to diastolic dysfunction [56]. In addition, *IGFBP7* also modulates the pro-angiogenic properties of signalling factors such as *VEGF-A* and *IGF* [57]. Together with *CP*, *IGFBP7’s* upregulation suggests a broader disruption in placental vascular function, potentially through inflammation-mediated angiogenic imbalance.

### Angiogenesis and vascular remodelling

Although late-onset preeclampsia is not as strongly associated with placental vascular dysfunction as early-onset preeclampsia, genes such as *CP*, *IGFBP7*, *CDH13*, *ROBO1*, *UNC5C*, *NRXN3*, and *ITGA1* suggest subtle changes in angiogenic pathways. These genes are involved in endothelial guidance and vascular patterning, which may contribute to localised or mild vascular defects. Ceruloplasmin (*CP*) is predominantly expressed in the syncytiotrophoblast, with its expression notably increased under hypoxic conditions such as those present in preeclampsia. This suggests a protective role for *CP* in mitigating oxidative stress within the placenta [58]. Similarly, the differential expression of vascular regulators such as *IGFBP7* and *CDH13* points to sex-specific differences in angiogenic regulation, a key aspect of placental development and preeclampsia pathophysiology. The increased *ITGA1* expression may reflect a compensatory response aimed at promoting angiogenesis and vascular remodelling, potentially in response to sub-optimal spiral artery modification and compromised placental perfusion in late-onset preeclampsia. While late-onset preeclampsia is associated with less extensive maternal spiral artery remodelling abnormalities than observed in early-onset PE and a morphologically normal placenta [6], these changes in gene expression may still reflect subtle structural alterations that compromise placental function.

### Placental gene expression and cardiovascular disease associations

We find that many genes that are most highly expressed in preeclamptic male-bearing placentas compared to controls are also linked with cardiovascular function. Provocatively, in investigating gene expression across human tissues in the GTEx portal (https://www.gtexportal.org/home/), we observe that three genes that are more highly expressed in preeclamptic male-bearing placentas are also extremely highly expressed in adult artery: *IGFBP7*, *ITGA1*, and *VCAN* [59]. Further, GWAS studies have identified mutations in *IGFBP7* associated with diastolic blood pressure [60], cardiac arrest [61] and gestational diabetes [62], mutations in *ITGA1* associated with platelet count [63], lymphocyte count [61], and LDL measurement [61], and mutations in *VCAN* associated with monocyte count [64], triglyceride level [65, 66], and HDL measurements [67].

Importantly, other differentially expressed genes have previously had GWAS hits in them associated with cardiac function and conditions. Mutations in *CP* are associated with angiogenesis [68], *CDH13* mutations are associated with atherosclerosis [69, 70] and cardiovascular disease [71], *COL6A3* mutations are associated with ischemic stroke [72], myocardial infarction and hypertension [45], and descending and ascending aortic diameter [73], *ELOVL6* mutations are associated with monocyte and platelet count [74], platelet count during second trimester, delivery, and postpartum [75] and thrombocytopenia [76], and *NR3CD* has been associated with hypertension [77]. Finally, given the concerns about the developmental origins of disease, it is provocative that two of the genes that show differential expression between preeclamptic and control placentas in males, *COL6A3* [78] and *ROBO1* [79, 80], also have been associated with risk for Alzheimer’s disease, which is now believed to have a vascular component [81].

### Hormonal and metabolic pathways

Three genes identified as downregulated in our analysis, *NR3C2*, *CSHL1*, and *ALPP*, are implicated in hormonal and metabolic pathways. *NR3C2*, encodes the mineralocorticoid receptor (MR, also known as the Nuclear Receptor Subfamily 3 Group C Member 2), and is a key regulator of sodium homeostasis and blood pressure. Abnormalities in *NR3C2* effector mechanisms, particularly through maternal sodium (Na+) retention, have been shown to play a role in preeclampsia aetiology [82]. In our study, *NR3C2* expression was downregulated specifically in male-bearing placentas from preeclamptic pregnancies, suggesting a potential mechanism of *NR3C2* dysregulation distinct from the genetic variants described in previous literature. Functional polymorphisms of *NR3C2* have been associated with early-onset hypertension in a Spanish population study [83] and pregnancy-exacerbated early-onset hypertension with down regulation of MR in maternal mononuclear leucocytes [84]. However, without matched data from maternal mononuclear leucocytes, it is difficult to relate these findings to our study. The sex-specific dysregulation of *NR3C2*, independent of polymorphism-induced protein changes, which we are unable to measure, may reflect a placenta-intrinsic mechanism relevant to male-specific late-onset preeclampsia and highlights the potential importance of this mechanism in understanding preeclampsia subtypes and sex differences in pregnancy outcomes.

*CSHL1* is a placental gene closely related to the human placental lactogen family, encoded in the *GH-CSH* gene cluster. It is structurally similar to chorionic somatomammotropin hormones (*CSH1*/*CSH2*), which contribute to metabolic adaptation during pregnancy. Despite this, *CSHL1* itself has until recently not been implicated as a key driver or biomarker in preeclampsia or other metabolic disorders of pregnancy such as gestational diabetes mellitus. However, this may be largely due to the lack of research as Hao et al. [85] found that circulating levels of placenta-related proteins, including *CSHL2*, have strong potential as early serum biomarkers, with altered levels observed in women who later develop preeclampsia. The observed reduction in *ALPP* mRNA despite previous reports of increased protein levels in preeclamptic placentas underscores a well-documented transcript-protein disconnect in preeclampsia, potentially reflecting adaptive mechanisms such as enhanced protein stability or translational efficiency [86–88]. Such regulation may enable the placenta to sustain critical metabolic and transport functions in the face of transcriptional downregulation and oxidative stress.

### Extracellular matrix (ECM) and cell-cell adhesion

Several upregulated genes in male-bearing PE placentas, including *VCAN*, *COL6A3*, *PCOLCE*, *NID2*, *PXDN*, *CHSY3* and *ADAMTS12* highlight disruption in the ECM, which is central to placental structural integrity and trophoblast invasion, and a range of essential signalling and developmental processes. *VCAN*, a large proteoglycan integral to ECM organisation, modulates cell adhesion, migration, and proliferation. Its dysregulation can influence trophoblast behaviour and potentially contribute to the low-grade inflammatory environment characteristic of preeclampsia. The upregulation of *COL6A3*, encoding the α3 chain of collagen VI, is particularly noteworthy as it was specifically observed in preeclampsia males compared to control males, despite previous studies finding no dysregulation in preeclampsia [89, 90]. While *COL6A3* upregulation has been associated with increased placental collagen in maternal smoking [91], its broader implications are significant: Collagen VI influences fibronectin network architecture [92], cell proliferation [93], and cell survival through activation of focal adhesion proteins [94]. *PCOLCE*, which enhances procollagen C-proteinase activity, plays a crucial role in ECM assembly through facilitation of fibrillar collagen maturation. Its altered expression may compromise ECM integrity and structural support necessary for proper placental development. Similarly, NID2, encoding the basement membrane glycoprotein nidogen-2, bridges laminin and type IV collagen [95] and *PXDN* enzymatically crosslinks type IV collagen, thereby stabilising basement membrane architecture [96]. Alterations in the expression of either gene could impair trophoblast anchoring to maternal decidua, potentially destabilising cellular interactions and placental architecture. While direct empirical support in late-onset preeclampsia is still emerging, we hypothesise that dysregulation of *ADAMTS12*, an ECM remodelling enzyme, may impair tissue remodelling, trophoblast invasion, and spiral artery remodelling, even in the absence of overt vascular pathology, and this ECM dysfunction may be relevant in late-onset preeclampsia [97]. Collectively, these ECM alterations may contribute to impaired placental remodelling and trophoblast invasion which although more characteristic of early-onset preeclampsia, may also prove to be an important characteristic of late-onset preeclampsia.

### Cytoskeletal and cellular dynamics

Differentially expressed genes involved in cytoskeletal organisation, such as *DBN1* and *ANK2*, suggest potential alterations in trophoblast motility and invasion. Proper cytoskeletal dynamics are crucial for trophoblast migration, and dysregulation may compromise their invasive capacity [98]. Such cytoskeletal disruptions are associated with impaired trophoblast function and may contribute to trophoblast dysfunction commonly observed in preeclampsia, impacting placental development and maternal–fetal interface [99, 100].

### Signalling pathways

Signalling-related genes, including *SGK2*, *PXDN*, and *CHSY3*, were also altered in male-bearing placentas from late-onset preeclamptic pregnancies. While *SGK1* was the predominantly expressed isoform in our placental samples (Supplementary Tables 2 and 3), both *SGK2* and *SGK3* were also detected, albeit at lower levels. Notably, *SGK2* expression was upregulated in placentas from male-bearing pregnancies affected by late-onset preeclampsia. Given that *SGK1* has been implicated in renal sodium retention and associated with hypertension, these findings highlight the need for continued investigation into the potential roles of *SGK2* in both placental function and maternal cardiovascular health [101].

### Gene set enrichment analysis reveals RNA processing and nuclear transport mechanisms

The enrichment of biological processes reveals a significant disruption in RNA metabolism, intracellular transport, and placental development, offering insights into the molecular underpinnings of placental dysfunction. Processes related to RNA splicing (Supplementary Table 8) (*GOBP_RNA_SPLICING_VIA_TRANSESTERIFICATION_REACTIONS*, *GOBP_RNA_SPLICING*, and *GOBP_NEGATIVE_REGULATION_OF_RNA_SPLICING*) and RNA transport (*GOBP_RNA_EXPORT_FROM_NUCLEUS*, *GOBP_MRNA_EXPORT_FROM_NUCLEUS*, and *GOBP_RNA_LOCALIZATION*) highlight an overarching perturbation in RNA processing and localisation, which may compromise transcript stability and the efficiency of protein translation. These disruptions are coupled with altered vesicle targeting (*GOBP_VESICLE_TARGETING*), reflecting broader deficits in intracellular trafficking that could further impact cellular compartmentalization and signalling. Of particular interest is the enrichment of *GOBP_EMBRYONIC_PLACENTA_MORPHOGENESIS*, underscoring the biological relevance of these molecular changes to placental development and structural integrity. The inclusion of *GOBP_NUCLEOBASE_BIOSYNTHETIC_PROCESS* suggests a compensatory upregulation of nucleotide metabolism, potentially reflecting cellular stress responses to increased RNA turnover or mis regulation. Together, these findings suggest that the observed transcriptomic alterations may underlie impaired trophoblast function, RNA-dependent regulatory mechanisms, and placental remodelling, contributing to compromised placental efficiency and associated clinical outcomes.

### Cell type proportion prediction

Cell-type deconvolution analysis revealed distinct immunological profiles between male and female late-onset preeclampsia placentas (Fig. 4). In late-onset preeclamptic pregnancies, male-bearing placentas showed increased proportions of CD14 + Monocytes and CD8 + Activated T Cells, while female-bearing placentas exhibited elevated levels of Fetal Nucleated Red Blood Cells and Maternal Plasma Cells. These sex-specific differences in immune cell composition suggest divergent inflammatory responses in late-onset preeclampsia that may contribute to the observed transcriptional differences. The correlation between immune cell proportions and gene expression patterns further supports the notion that cellular composition plays a crucial role in altered placental gene expression profiles that are often associated with preeclampsia, although it remains unclear whether these differences drive the pathophysiology or arise as a response to systemic maternal factors.

### Functional analysis of gene correlation modules

Our gene co-expression analysis revealed distinct co-expression modules that provide mechanistic insights into the molecular pathology of preeclampsia, capturing both cellular composition and intrinsic gene expression changes within the placenta. Modules positively correlated with late-onset preeclampsia, particularly the orange and red modules, showed strong associations with fetal mesenchymal stem, cell proportions, implicating placental stromal cell activation in preeclamptic pathology, alongside the traditionally emphasised trophoblast dysfunction. Notably, enrichment for extracellular matrix organisation and cell-substrate adhesion in the orange module, which contains 61 of the 150 differentially expressed genes, supports the hypothesis that aberrant stromal remodelling and defective cell-extra cellular matrix interactions contribute to the pathophysiology of preeclampsia. Although less directly linked, terms such as *striated muscle hypertrophy* and *sensory system development* may reflect broader roles of cytoskeletal remodelling and morphogenic signaling pathways repurposed in placental mesenchymal cells. The red module’s enrichment for vasoconstriction and circulatory regulation aligns with the hallmark vascular dysregulation of preeclampsia, while the orange module’s involvement in extracellular matrix organisation and developmental processes suggest impaired stromal remodelling. In contrast, the pink, purple and green modules were negatively associated with preeclampsia, pointing towards disrupted pathways in healthy placental function. The light blue module’s strong correlation with syncytiotrophoblast abundance and enrichment in fundamental metabolic processes underscores the impaired metabolic adaptation characteristic of preeclamptic placentas. Meanwhile, the dark blue module’s correlation with gestational age and enrichment for epigenetic regulatory processes suggests a potential link between reduced chromatin remodelling and the placental developmental immaturity observed in this disorder. Together these findings highlight the value of network-level transcriptomic analysis in disentangling the complex functional and cellular alterations that underpin preeclampsia.

### Limitations of the study

A notable limitation of this study is the potential confounding effect of cell type proportions on our transcriptional analyses. The differential gene expression and WGCNA findings may partially reflect changes in cellular composition rather than cell type-specific transcriptional regulation. This is particularly relevant given the significant differences we observed in immune cell populations between preeclamptic and control placentas, specifically in CD14 + Monocytes and CD8 + Activated T Cells in male samples, and Fetal Nucleated Red Blood Cells and Maternal Plasma Cells in female samples. The Pearson’s correlation analyses between gene expression and predicted cell type proportions should be interpreted with caution, as they describe associations only and cannot distinguish whether increased expression reflects true upregulation within a cell type or a higher abundance of that cell type in the tissue. Although these correlations help to contextualise these relationships, they do not provide definitive evidence of cell type–specific transcriptional regulation. Future studies employing single-cell RNA sequencing will be important to distinguish transcriptional changes occurring within specific cell types from those driven by shifts in cellular composition. Moreover, experimental validation using qPCR, immunohistochemistry, purified primary cell analyses, or spatial transcriptomics will be essential to confirm differential expression and clarify the relative contributions of cellular composition versus intrinsic transcriptional regulation in the pathophysiology of late-onset preeclampsia.

## Conclusion

The absence of significant differential expression in female-bearing placentas with and without late-onset preeclampsia is particularly intriguing and warrants further investigation. This finding suggests that female-bearing placentas may either be more resilient to the pathological processes of late-onset preeclampsia or employ different adaptive mechanisms that are not captured by bulk transcriptional analysis. The observation of cell type proportion changes in female-bearing placentas, despite the lack of differential gene expression, supports the latter hypothesis and highlights the complexity of placental adaptation to pathological conditions.

These findings have important implications for our understanding of preeclampsia pathophysiology and may inform future research directions. The marked sexual dimorphism observed in placental transcriptional profiles associated with late-onset preeclampsia suggests that sex-specific investigative strategies may be necessary. Furthermore, the distinct immune cell profiles between male- and female-bearing placentas indicate that future research might need to be tailored according to fetal sex.

A key observation from the placental gene expression data is that the subset of genes differentially expressed between preeclamptic and uncomplicated male-bearing pregnancies did not show comparable changes in female-bearing pregnancies. Instead, placentas from female-bearing pregnancies, whether preeclamptic or control, displayed expression profiles more similar to those of male controls. This suggests that the transcriptional differences associated with late-onset preeclampsia are specific to male-bearing placentas, whereas the female-bearing placentas lack a comparable signal.

Taken together, our findings suggest that gene expression changes observed in male-bearing placentas during preeclampsia may reflect a persistent mechanism that results in an effect on maternal vascular stress. The absence of similar changes in female-bearing placentas raises questions about potential sex-specific resilience mechanisms. These results highlight important directions for future research, including the use of single-cell RNA sequencing to resolve cell type-specific responses, investigation into the molecular basis of female placental resilience, and longitudinal studies to elucidate the temporal dynamics of sex-specific placental adaptation to hypertensive stress. Such work may ultimately uncover novel protective pathways with therapeutic potential.

## Supplementary Information

Supplementary Material 1

Supplementary Material 2

Supplementary Material 3

Supplementary Material 4

Supplementary Material 5

Supplementary Material 6

Supplementary Material 7

Supplementary Material 8

Supplementary Material 9

Supplementary Material 10

Supplementary Material 11

Supplementary Material 12

Supplementary Material 13

Supplementary Material 14

## Data Availability

The datasets analysed during the current study are available in the NCBI GEO repository, using accession number GSE306864 [https://www.ncbi.nlm.nih.gov/geo/query/acc.cgi? acc=GSE306864](https:/www.ncbi.nlm.nih.gov/geo/query/acc.cgi? acc=GSE306864).
