# Supplementary figures and images for: Sex-specific placental transcriptome alterations in late-onset preeclampsia reveal male-biased immune and metabolic dysregulation
Source: Biol Sex Differ. 2025 Dec 24;17:8. doi: 10.1186/s13293-025-00781-w (PMC12809948; doi:10.1186/s13293-025-00781-w)

# Cluster Dendrogram

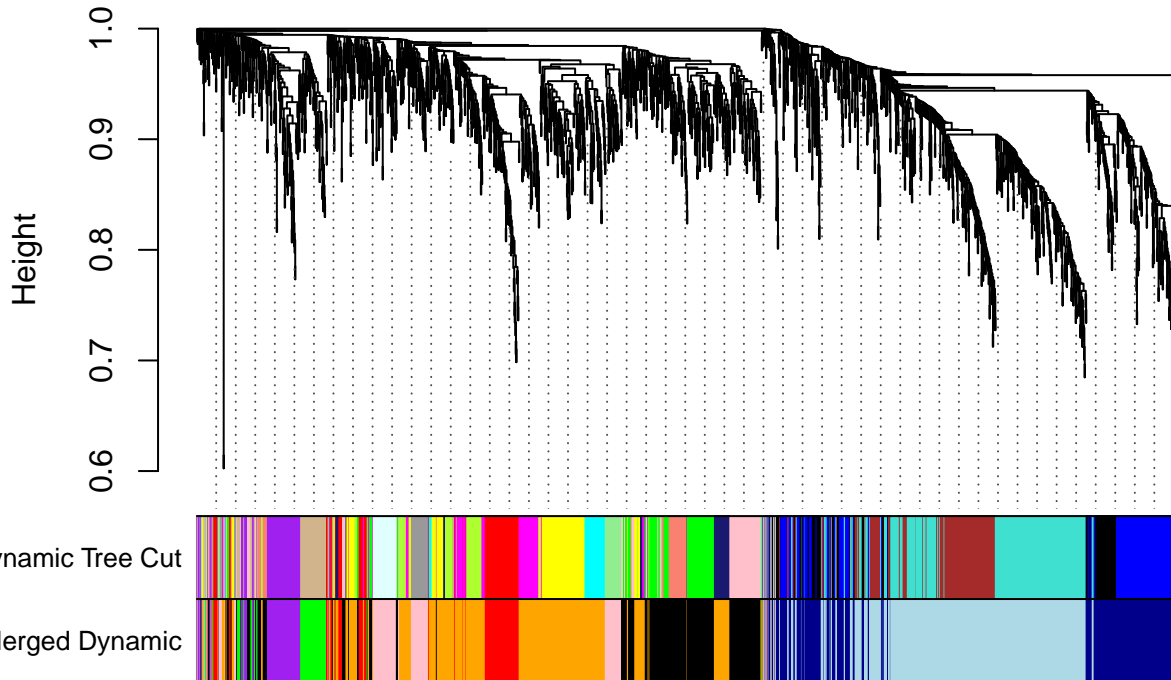

Supplement: Supplementary file 1 — Supplementary Material 1 [file 13293_2025_781_MOESM1_ESM.pdf]

**CIBERSORTx Adjusted Signature Matrix (scaled by row)**

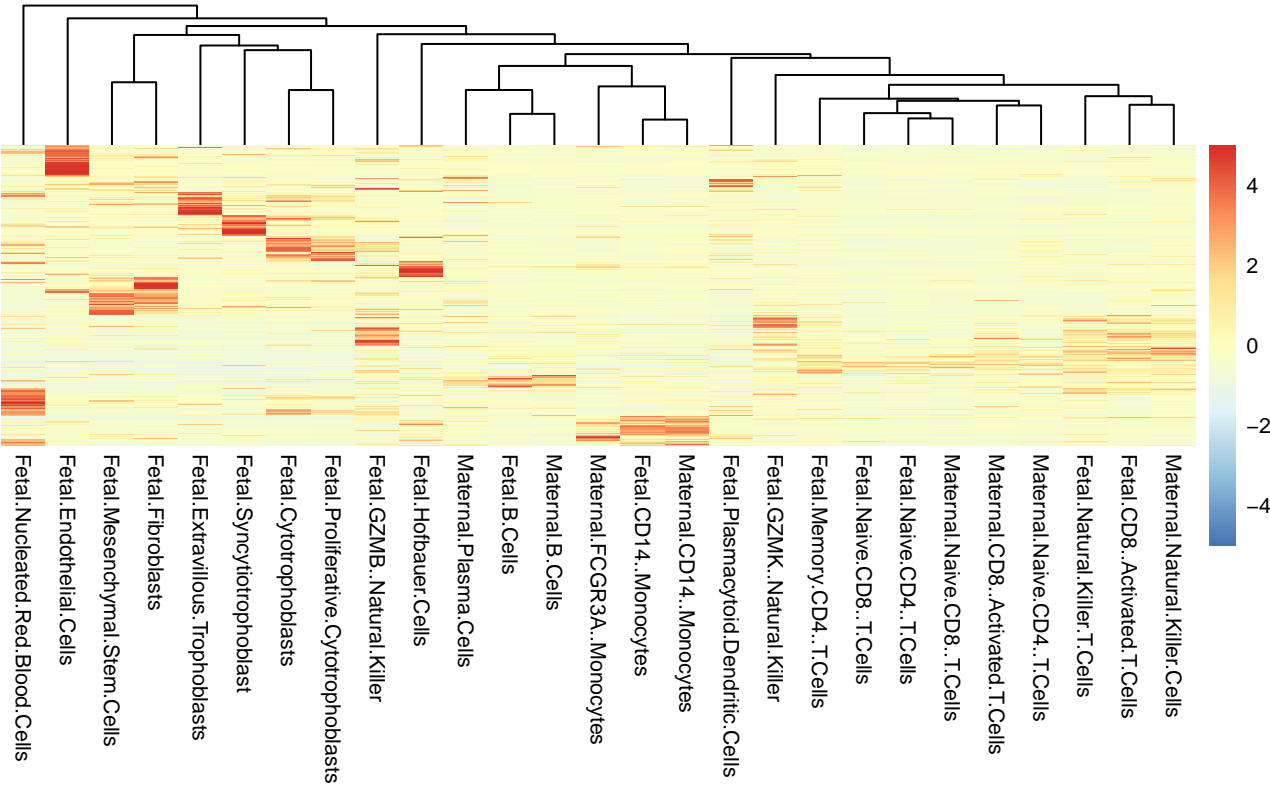

Supplement: Supplementary file 2 — Supplementary Material 2 [file 13293_2025_781_MOESM2_ESM.pdf]

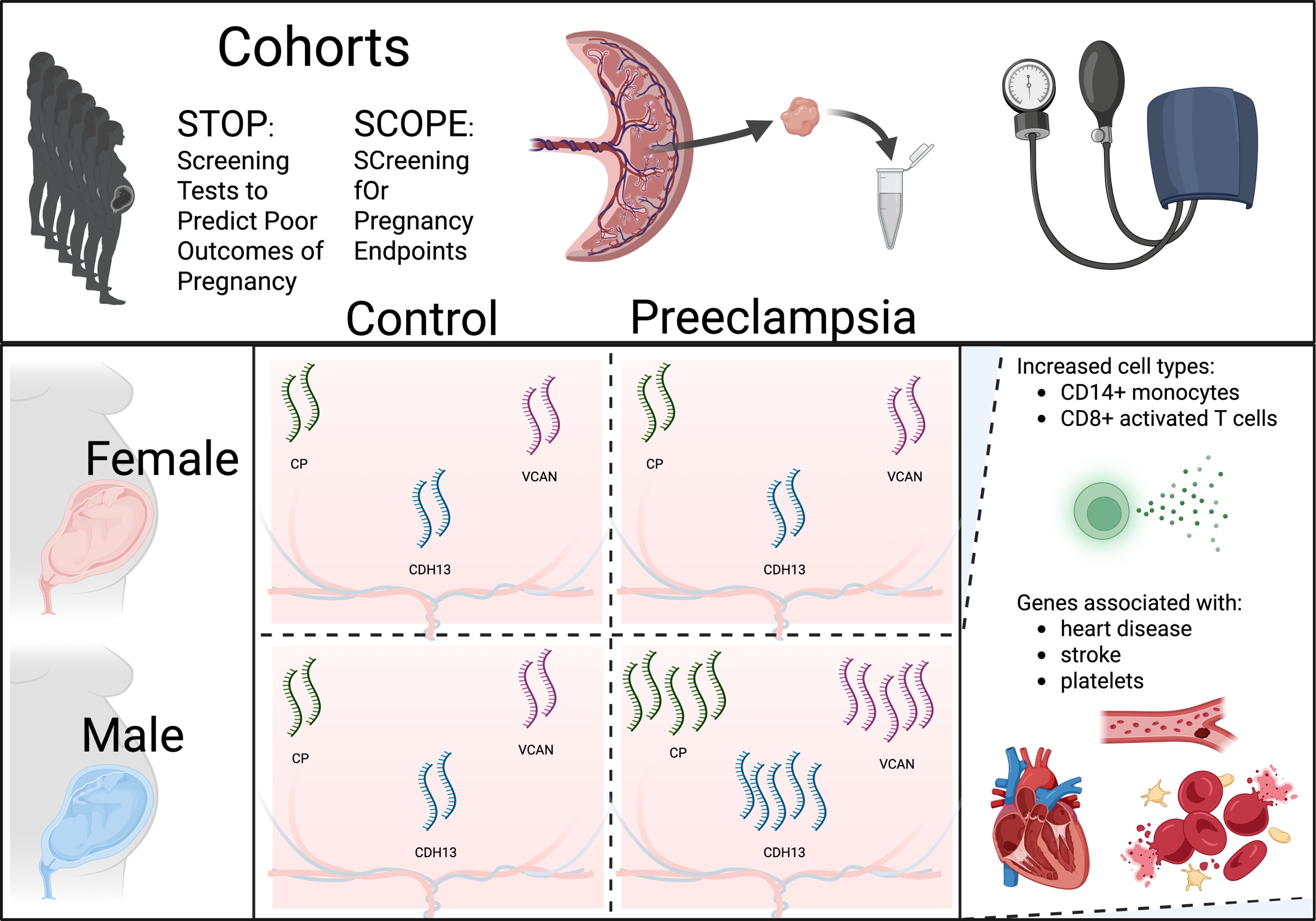

Supplement: Supplementary file 11 — Supplementary Material 11 [file 13293_2025_781_MOESM11_ESM.jpg]
