# Supplementary material for: Sex-specific placental transcriptome alterations in late-onset preeclampsia reveal male-biased immune and metabolic dysregulation
Source: Biol Sex Differ. 2025 Dec 24;17:8. doi: 10.1186/s13293-025-00781-w (PMC12809948; doi:10.1186/s13293-025-00781-w)
Supplement: Supplementary file 3 — Supplementary Material 3 [file 13293_2025_781_MOESM3_ESM.docx]

Supplementary Note 1: BAM file deduplication for RNA-Seq data

Although deduplication is not standard practice in RNA sequencing workflows, due to the expectation that highly expressed genes will naturally yield duplicate reads reflecting biological signal, we chose to remove duplicates from our aligned BAM files after observing evidence of technical duplication artefacts in our dataset.

Quality control using FASTQC (v 0.12) revealed unusually high levels of duplication in several samples. Visual inspection of aligned reads in IGV further supported the presence of PCR duplicates, with multiple instances of identical reads mapping to the same genomic coordinates. In some cases, nearly every read or every second read appeared duplicated, raising concerns about quantification accuracy.

To evaluate the impact of deduplication, we compared results before and after removing duplicates using a range of metrics:

- **Gene expression values** (in counts per million [CPM] and transcripts per million [TPM]) remained highly concordant between deduplicated and original datasets, with a slight and expected attenuation of the most highly expressed genes.
- **Library size** (total raw counts) was reduced in a consistent manner across samples (Figure 1).
- **Differential expression analysis** results (adjusted *p*-values and log fold changes) remained stable post-deduplication, with high concordance observed across the dataset (Figure 2 and 3).
- **Cell-type deconvolution using CIBERSORTx** improved after deduplication. Predicted gene expression aligned more closely with observed gene expression values in TPM, particularly for signature genes (Figure 4). In scatter plots comparing observed versus predicted expression, deduplication data showed tighter clustering around the identity line (X = Y), suggesting improved model accuracy.

These analyses suggest that PCR duplication in our dataset significantly impacted read counts and downstream analyses. We therefore elected to deduplicate all BAM files prior to quantification. While we acknowledge this approach may lead to minor underestimation of some highly expressed genes, our data indicate that the benefits, in terms of improved modelling and data quality, outweigh thus limitation in our context.


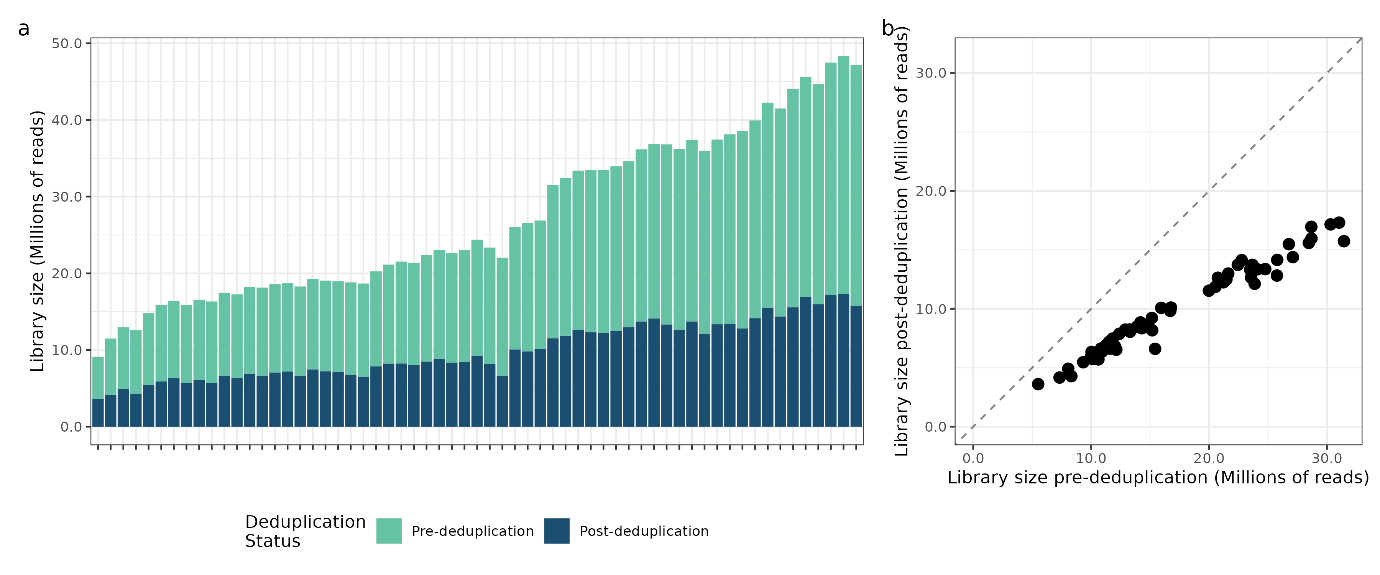


**Figure 1**. *Stacked bar chart (a) and scatterplot (b) of library size pre- and post-deduplication.* Total raw counts were reduced in a consistent manner across all samples with larger libraries diverging further from the identity line (X = Y) suggesting deduplication worked as expected with as larger libraries have a higher probability of containing duplicate sequences due to their size. As library size increases, the chance that some sequences appear multiple times grows substantially. This creates more opportunities for deduplication to remove redundant entries.


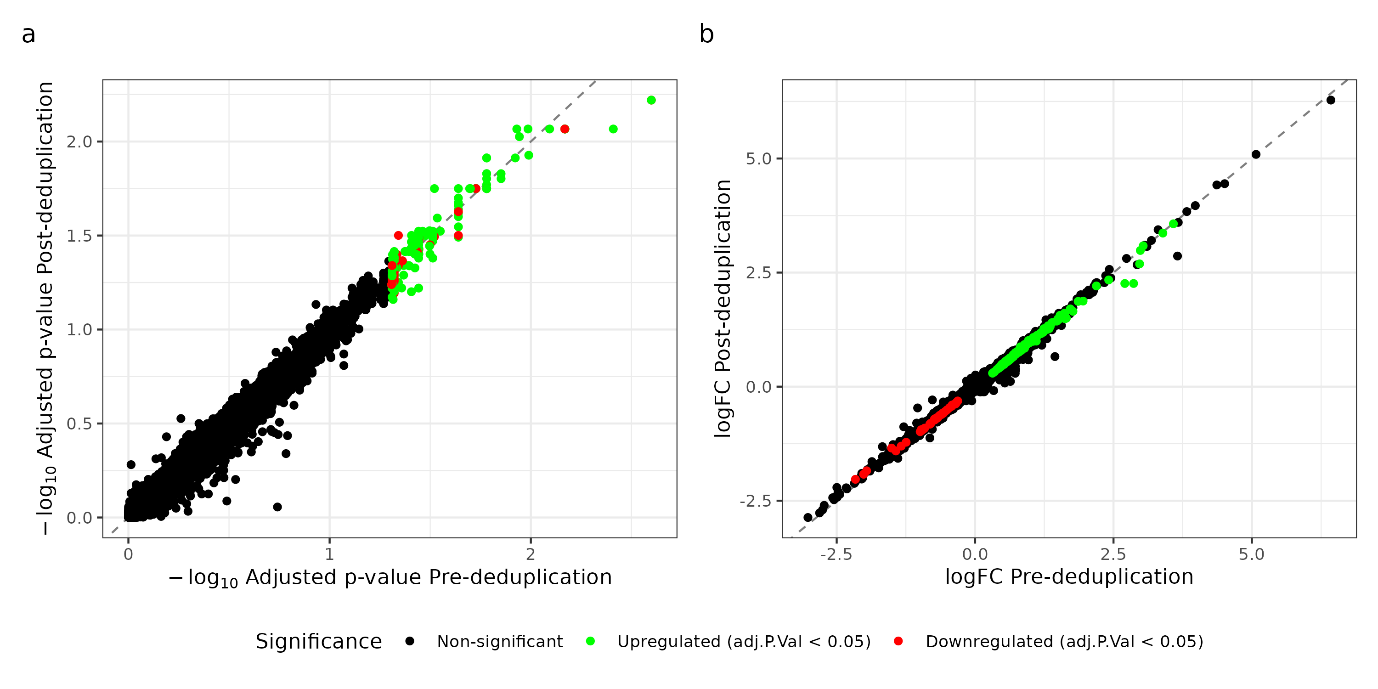


**Figure 2.** *Scatter plots comparing pre- and post-deduplication differential expression results from male placentas from preeclamptic and uncomplicated pregnancies*. (a) Adjusted p-values (-log_10_ transformed) and (b) log fold-changes are shown for each gene. Points are coloured by significance: black indicates non-significant genes, green indicates significantly upregulated genes (adjusted *p* < 0.05), and red indicates significantly downregulated genes (adjusted *p* < 0.05). The dashed line represents perfect concordance between pre- and post-deduplication results. There is strong overall agreement between both the adjusted *p-*values and log fold-change values, supporting the robustness of the deduplication step.


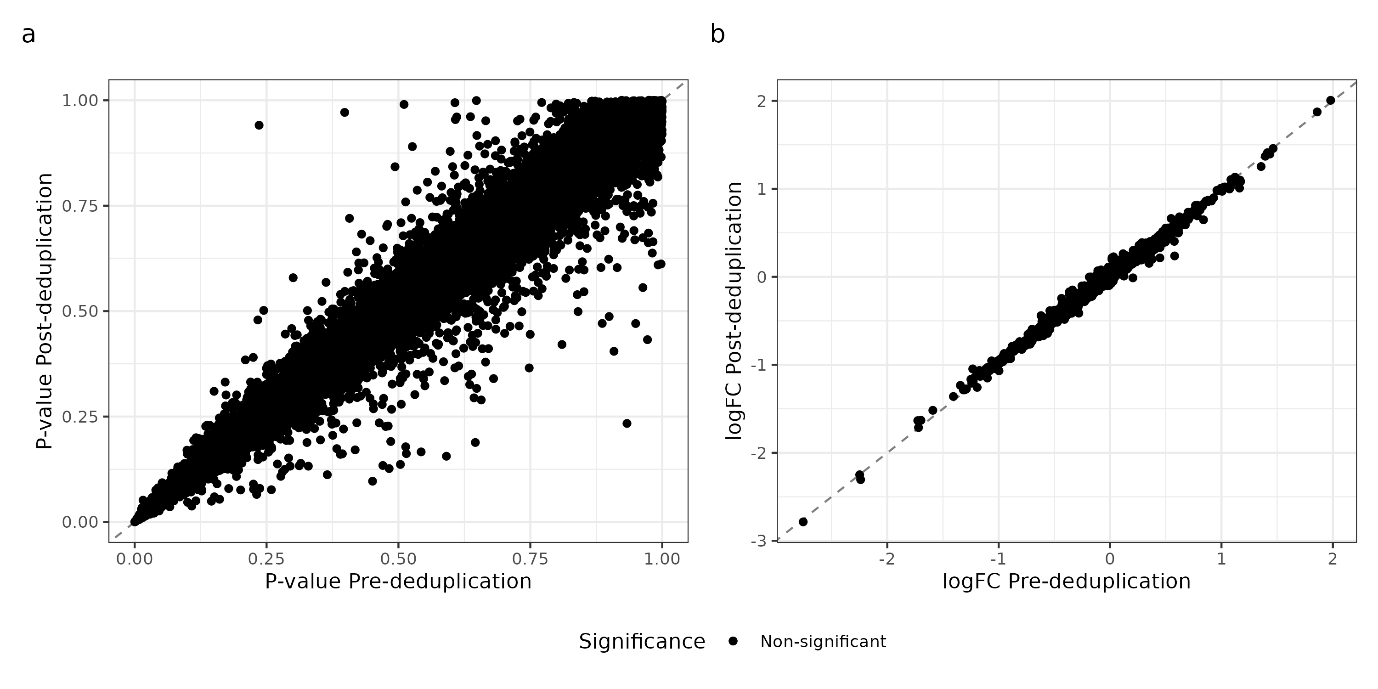


**Figure 3.** *Scatter plots comparing pre- and post-deduplication differential expression results from female placentas from preeclamptic and uncomplicated pregnancies.* (a) P- and (b) log fold-changes are shown for each gene. Points are coloured by significance: black indicates non-significant genes (no genes with an adjusted *p-*value < 0.05). The dashed line represents perfect concordance between pre- and post-deduplication results. There is reasonable agreement between *p*-values pre- and post-deduplication, though post-deduplication *p*-values tend to be lower than pre-deduplication values, likely due to improved signal-to-noise ratio after removing duplicates.


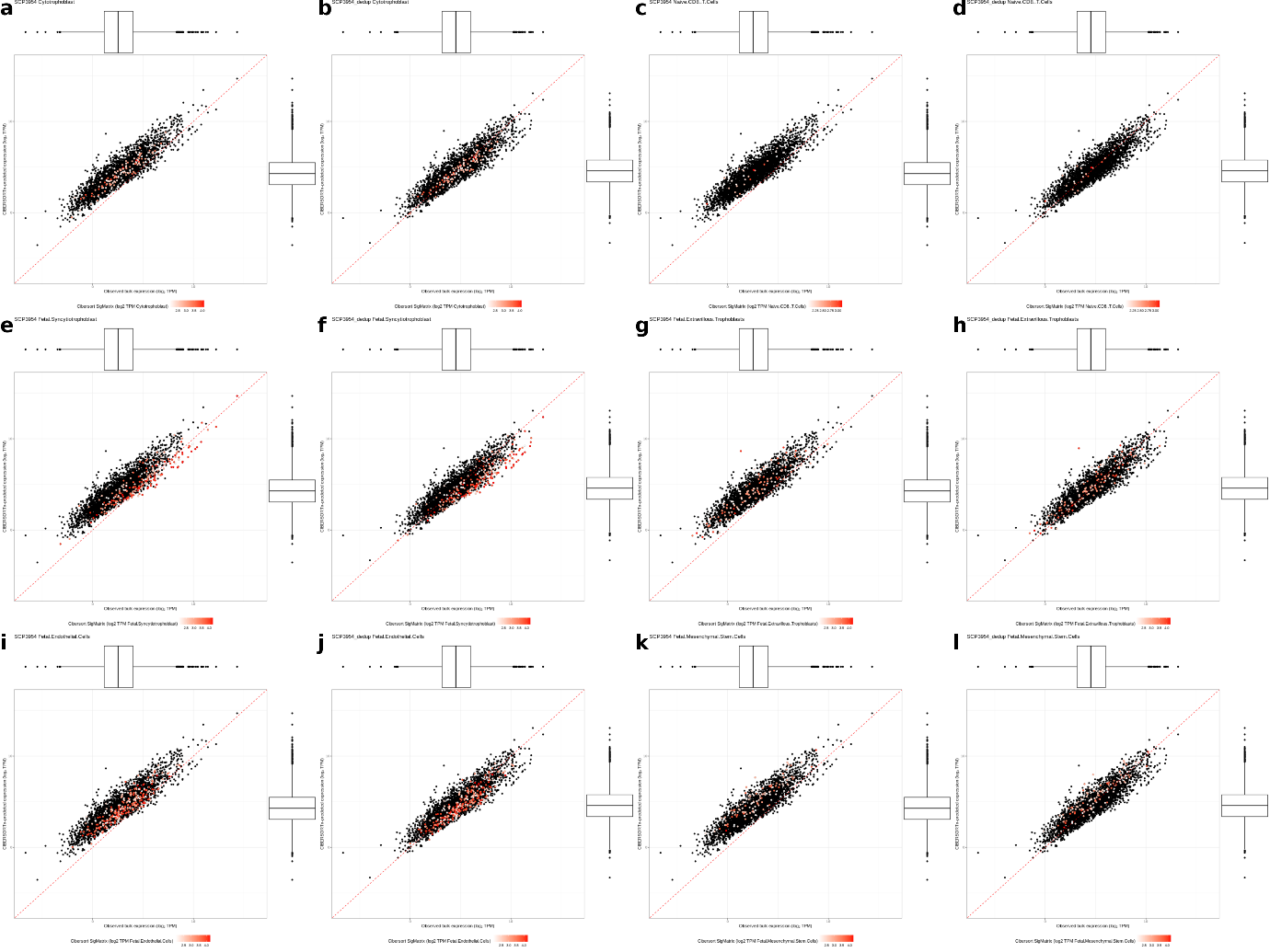


**Figure 4**. *Scatter plots comparing observed gene expression (log_2_ TPM, x-axis) and predicted gene expression (log_2_ TPM, y-axis) from CIBERSORTx for representative sample data (SCP3954, male, uncomplicated) across a subset of cell types.* Panels (a) and (b): Cytotrophoblast, (c) and (d): Naïve CD8 T Cells, (e) and (f): Fetal Syncytiotrophoblast, (g) and (h): Fetal Extravillous Trophoblast, (i) and (j): Fetal Endothelial Cells, (k) and (l): Fetal Mesenchymal Cells. Pre- and post-deduplication results are shown side by side for each cell type. In each case, signature genes are highlighted in red, with colour intensity scaled by expression level. Post-deduplication, signature genes showed stronger alignment with the identity line (X = Y, dotted red), indicating improved concordance between observed and predicted expression and suggesting enhanced CIBERSORTx model accuracy.
