# Supplementary material for: Sex-specific placental transcriptome alterations in late-onset preeclampsia reveal male-biased immune and metabolic dysregulation
Source: Biol Sex Differ. 2025 Dec 24;17:8. doi: 10.1186/s13293-025-00781-w (PMC12809948; doi:10.1186/s13293-025-00781-w)
Supplement: Supplementary file 4 — Supplementary Material 4 [file 13293_2025_781_MOESM4_ESM.pdf]

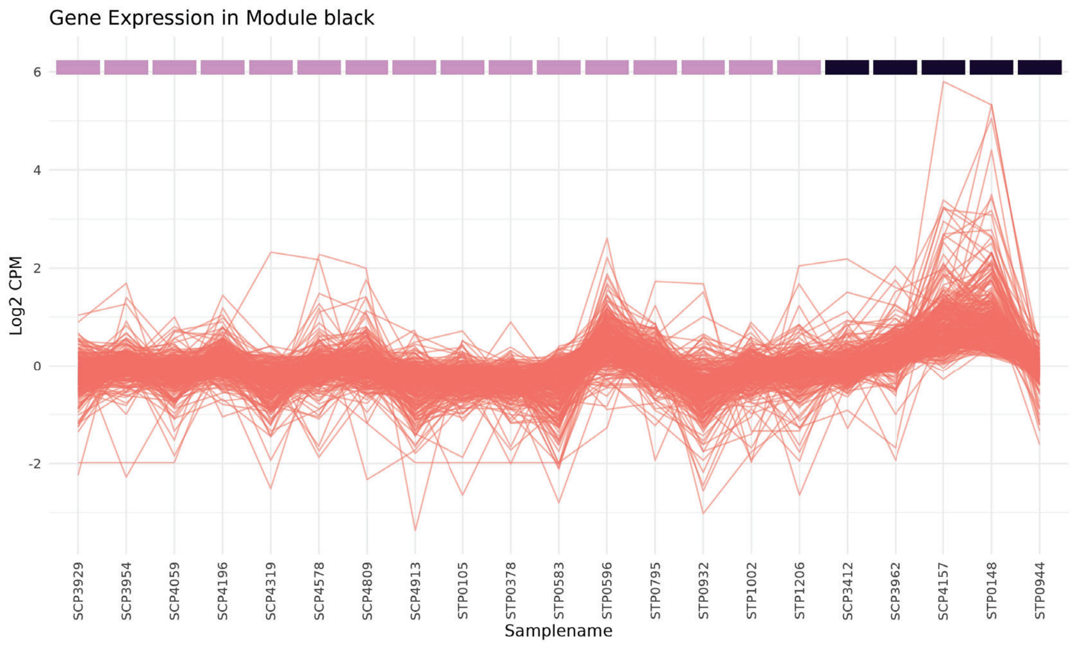
Gene Expression in Module black
Log2 CPM
6
4
2
0
-2
SCP3929
SCP3954
SCP4059
SCP4196
SCP4319
SCP4578
SCP4809
SCP4913
STP0105
STP0378
STP0583
STP0596
STP0795
STP0932
STP1002
STP1206
SCP3412
SCP3962
SCP4157
STP0148
STP0944
Samplename

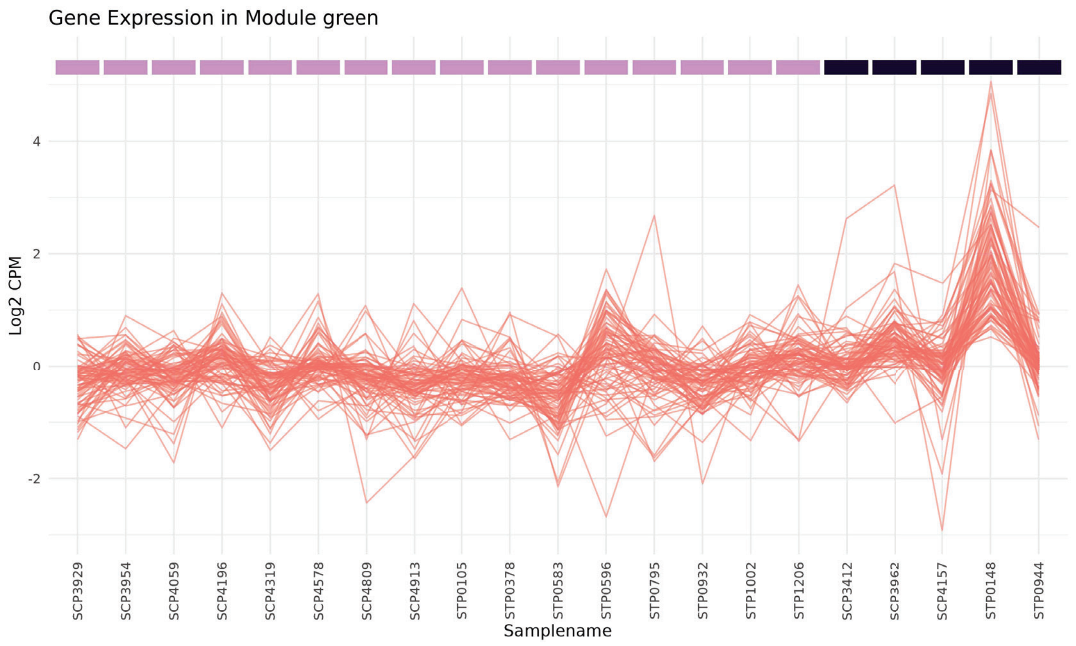
Gene Expression in Module green
Log2 CPM
4
2
0
-2
SCP3929
SCP3954
SCP4059
SCP4196
SCP4319
SCP4578
SCP4809
SCP4913
STP0105
STP0378
STP0583
STP0596
STP0795
STP0932
STP1002
STP1206
SCP3412
SCP3962
SCP4157
STP0148
STP0944
Samplename

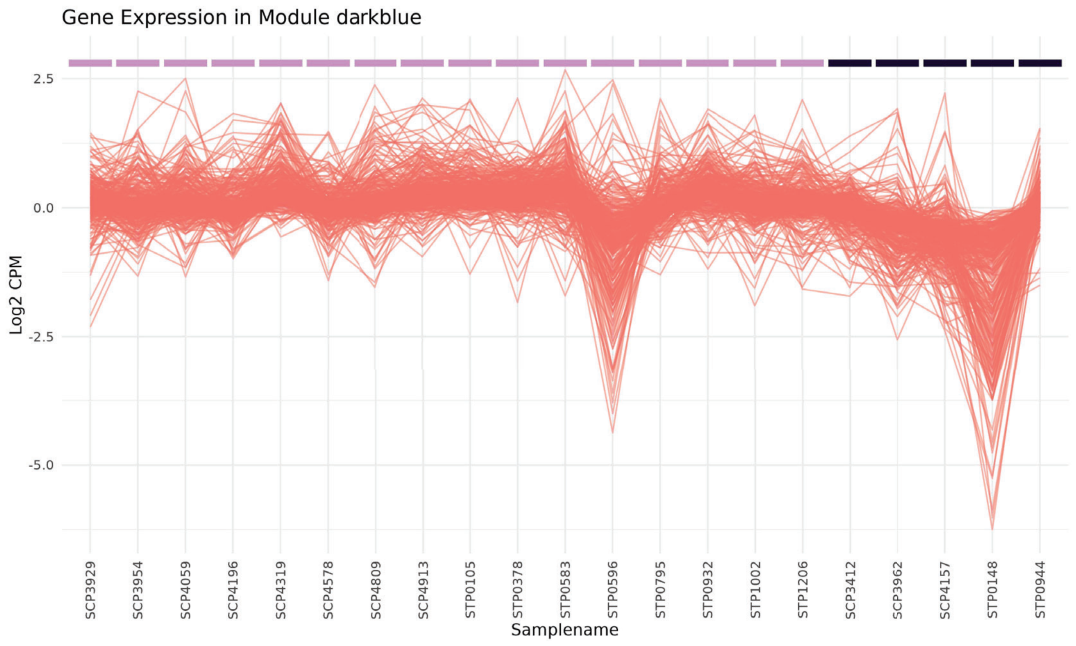
Gene Expression in Module darkblue
Log2 CPM
2.5
0.0
-2.5
-5.0
SCP3929
SCP3954
SCP4059
SCP4196
SCP4319
SCP4578
SCP4809
SCP4913
STP0105
STP0378
STP0583
STP0596
STP0795
STP0932
STP1002
STP1206
SCP3412
SCP3962
SCP4157
STP0148
STP0944
Samplename

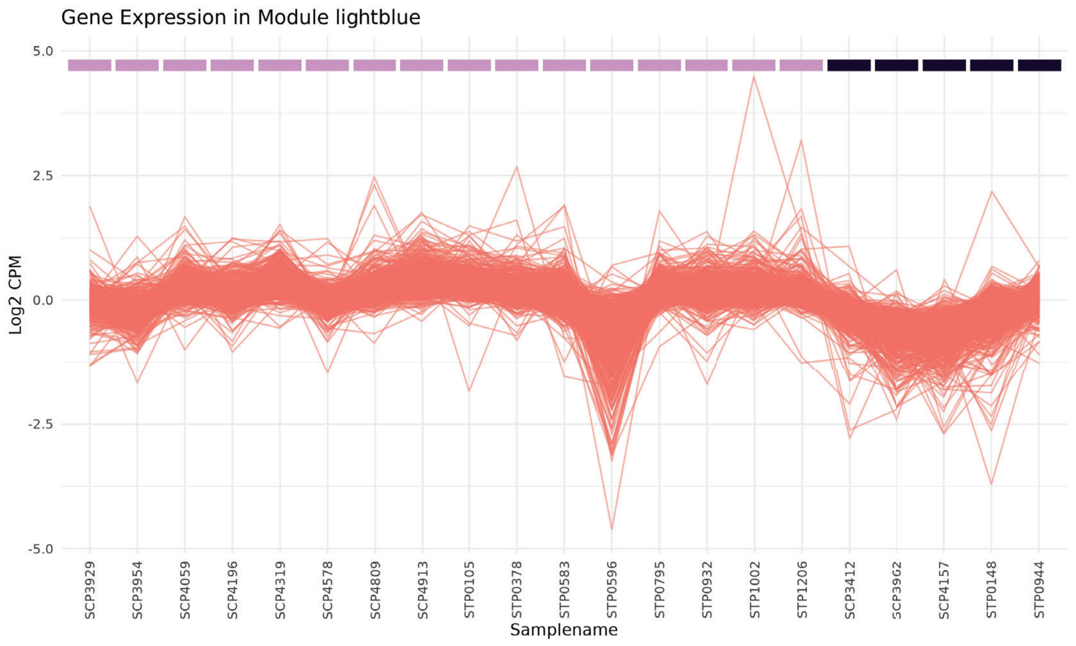
Gene Expression in Module lightblue
Log2 CPM
5.0
2.5
0.0
-2.5
-5.0
SCP3929
SCP3954
SCP4059
SCP4196
SCP4319
SCP4578
SCP4809
SCP4913
STP0105
STP0378
STP0583
STP0596
STP0795
STP0932
STP1002
STP1206
SCP3412
SCP3962
SCP4157
STP0148
STP0944
Samplename

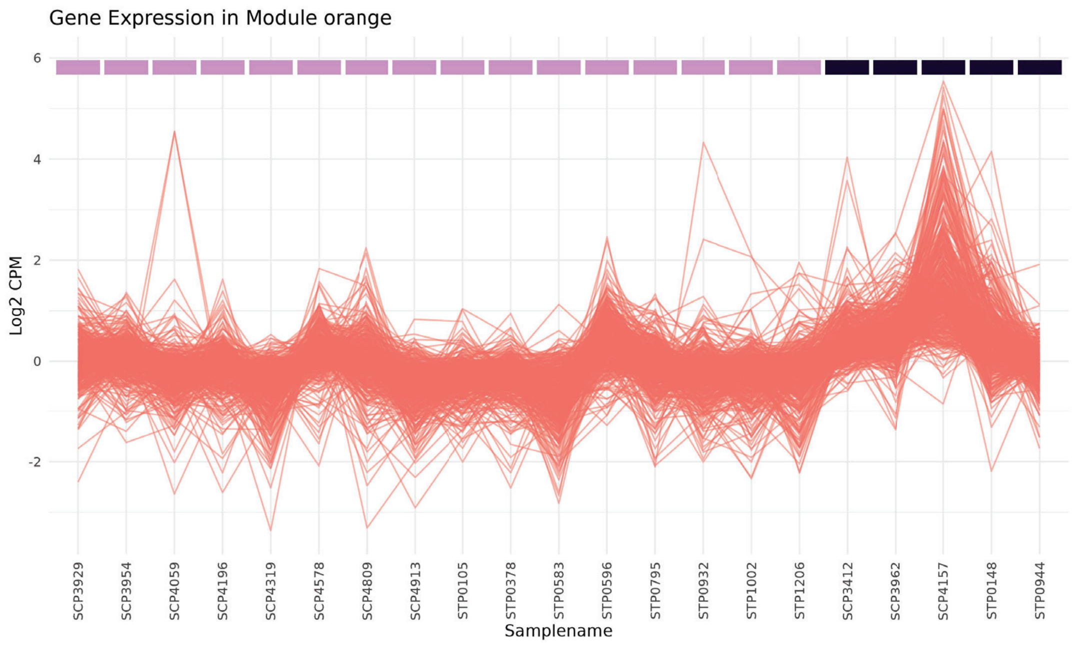
Gene Expression in Module orange
Log2 CPM
6
4
2
0
-2
SCP3929
SCP3954
SCP4059
SCP4196
SCP4319
SCP4578
SCP4809
SCP4913
STP0105
STP0378
STP0583
STP0596
STP0795
STP0932
STP1002
STP1206
SCP3412
SCP3962
SCP4157
STP0148
STP0944
Samplename

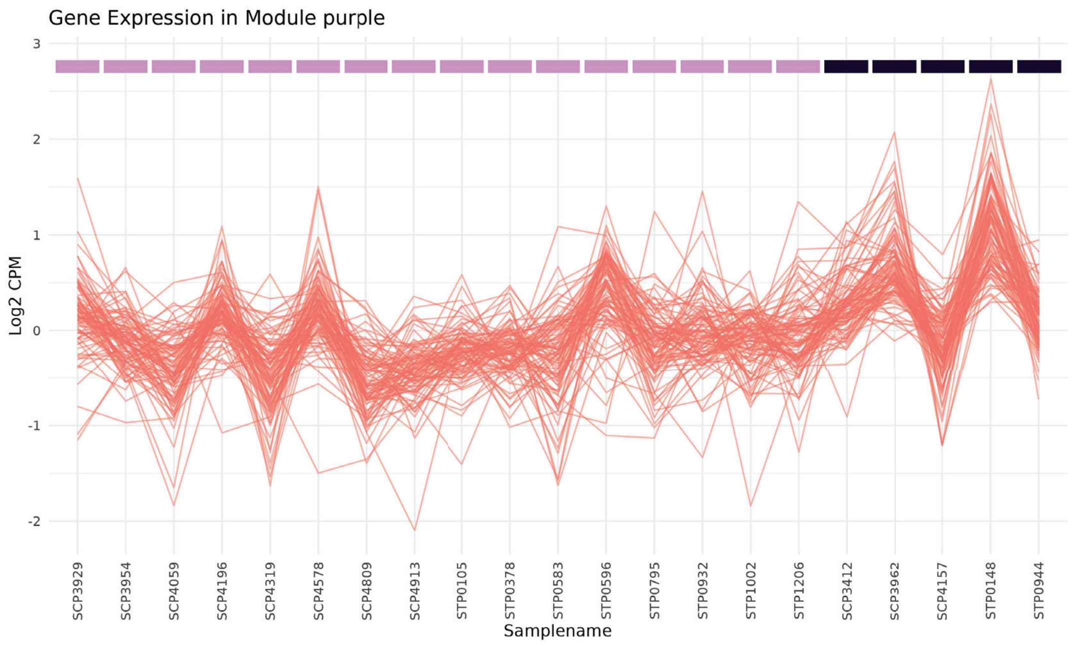
Gene Expression in Module purple
Log2 CPM
3
2
1
0
-1
-2
SCP3929
SCP3954
SCP4059
SCP4196
SCP4319
SCP4578
SCP4809
SCP4913
STP0105
STP0378
STP0583
STP0596
STP0795
STP0932
STP1002
STP1206
SCP3412
SCP3962
SCP4157
STP0148
STP0944
Samplename

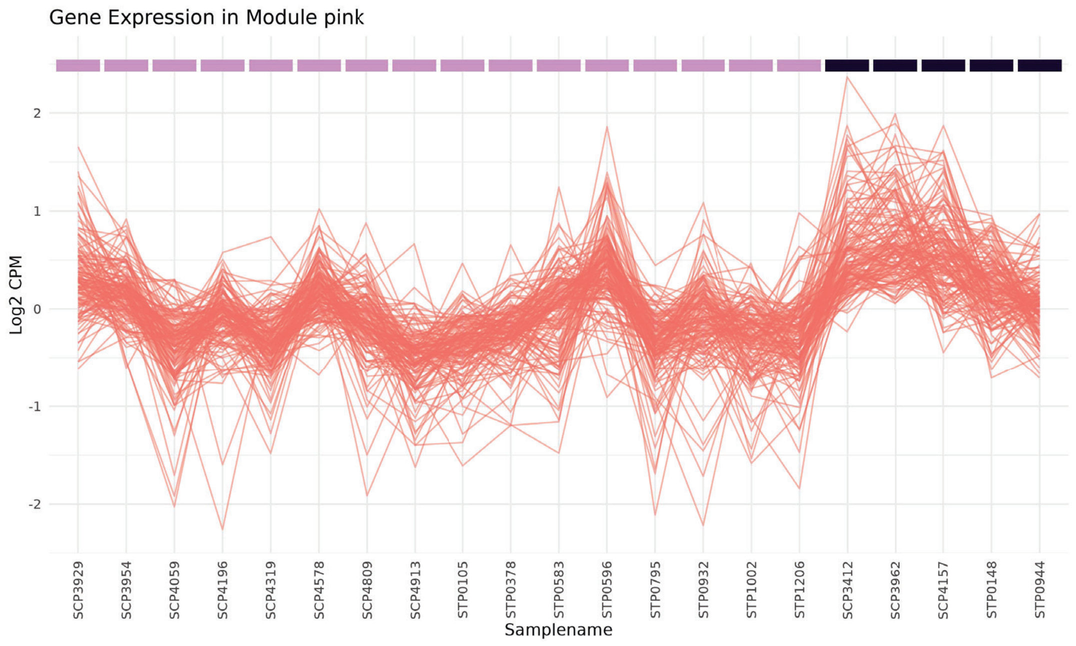
Gene Expression in Module pink
Log2 CPM
2
1
0
-1
-2
SCP3929
SCP3954
SCP4059
SCP4196
SCP4319
SCP4578
SCP4809
SCP4913
STP0105
STP0378
STP0583
STP0596
STP0795
STP0932
STP1002
STP1206
SCP3412
SCP3962
SCP4157
STP0148
STP0944
Samplename

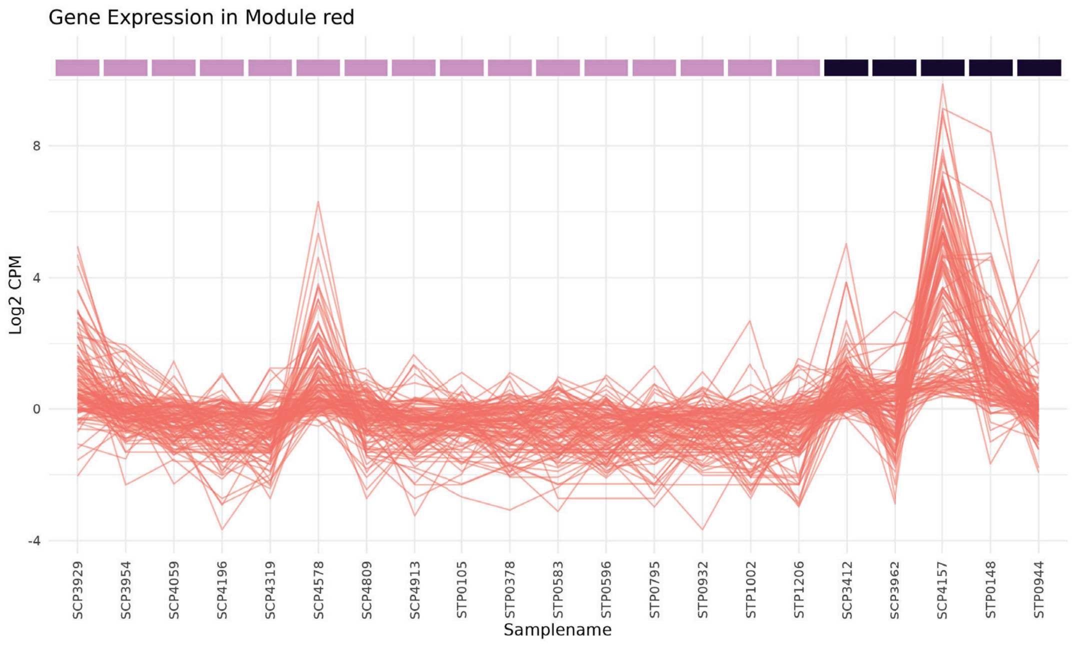
Gene Expression in Module red
Log2 CPM
8
4
0
-4
SCP3929
SCP3954
SCP4059
SCP4196
SCP4319
SCP4578
SCP4809
SCP4913
STP0105
STP0378
STP0583
STP0596
STP0795
STP0932
STP1002
STP1206
SCP3412
SCP3962
SCP4157
STP0148
STP0944
Samplename

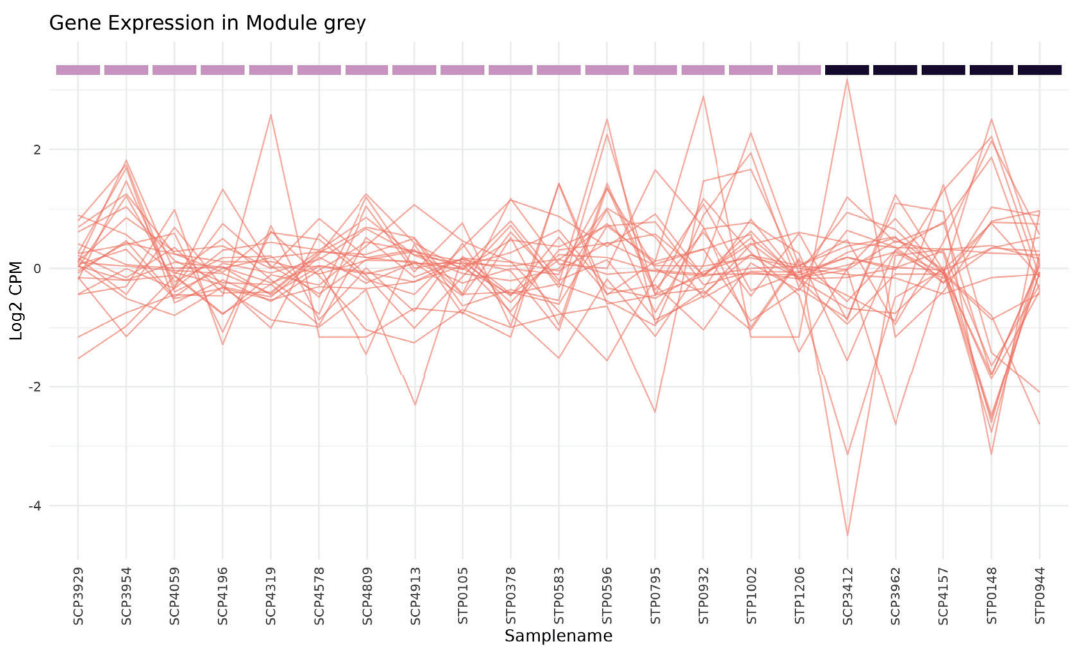

Gene Expression in Module grey
Log2 CPM
2
0
-2
-4
SCP3929
SCP3954
SCP4059
SCP4196
SCP4319
SCP4578
SCP4809
SCP4913
STP0105
STP0378
STP0583
STP0596
STP0795
STP0932
STP1002
STP1206
SCP3412
SCP3962
SCP4157
STP0148
STP0944
Samplename
