# Supplementary material for: Sex-specific placental transcriptome alterations in late-onset preeclampsia reveal male-biased immune and metabolic dysregulation
Source: Biol Sex Differ. 2025 Dec 24;17:8. doi: 10.1186/s13293-025-00781-w (PMC12809948; doi:10.1186/s13293-025-00781-w)
Supplement: Supplementary file 12 — Supplementary Material 12 [file 13293_2025_781_MOESM12_ESM.docx]

Supplementary Note 2. Rationale for the unadjusted regression model and results from alternative models

To identify gene expression differences associated with preeclampsia, we applied linear regression modelling using the *limma voom* pipeline, across all samples, with preeclampsia status as the primary explanatory variable. Subsequently, to identify gene expression differences that vary by fetal sex, we performed post-hoc analysis stratified by fetal sex. An initial multidimensional scaling plot (MDS) visualisation was used to identify the greatest source of variation in our data (Figure 1). We identified a clear separation by fetal sex on dimension 2, with no clear clustering based on cohort or outcome. Finding no clear bias in our data associated with maternal or fetal demographics we used a simple regression model without additional covariates in the main analysis. The decision to use the simple regression model was made after evaluating several covariate-inclusive models and assessing their impact on differential expression results.

Prior to the decision to use the simple regression model we explored several potential covariates including maternal age, maternal body mass index (BMI), smoking status and neonate birthweight, including custom birthweight centiles. Our investigation of these potential covariates found:

- **Maternal age**
  - In our cohort subsets all maternal ages fell within a low-risk range
  - There was no significant difference between maternal ages between the preeclamptic and uncomplicated pregnancies
- **Maternal BMI**:
  - There was no statistical difference between preeclamptic and uncomplicated pregnancies within the male comparison
  - There was a statistically significant difference between preeclamptic and uncomplicated pregnancies within the female comparison
  - To preserve the full biological signature associated with preeclampsia, including any contribution from maternal adiposity, BMI was excluded in the main analysis.
- **Smoking status**:
  - Too infrequent in the male subgroup to model robustly.
- **Birthweight**:
  - Excluded due to collinearity with gestational age.
  - Additionally, customised birth centiles, which adjust for maternal, paternal, and fetal characteristics, showed no significant group differences.

Maternal BMI, maternal age, and their combination were included in sensitivity models.


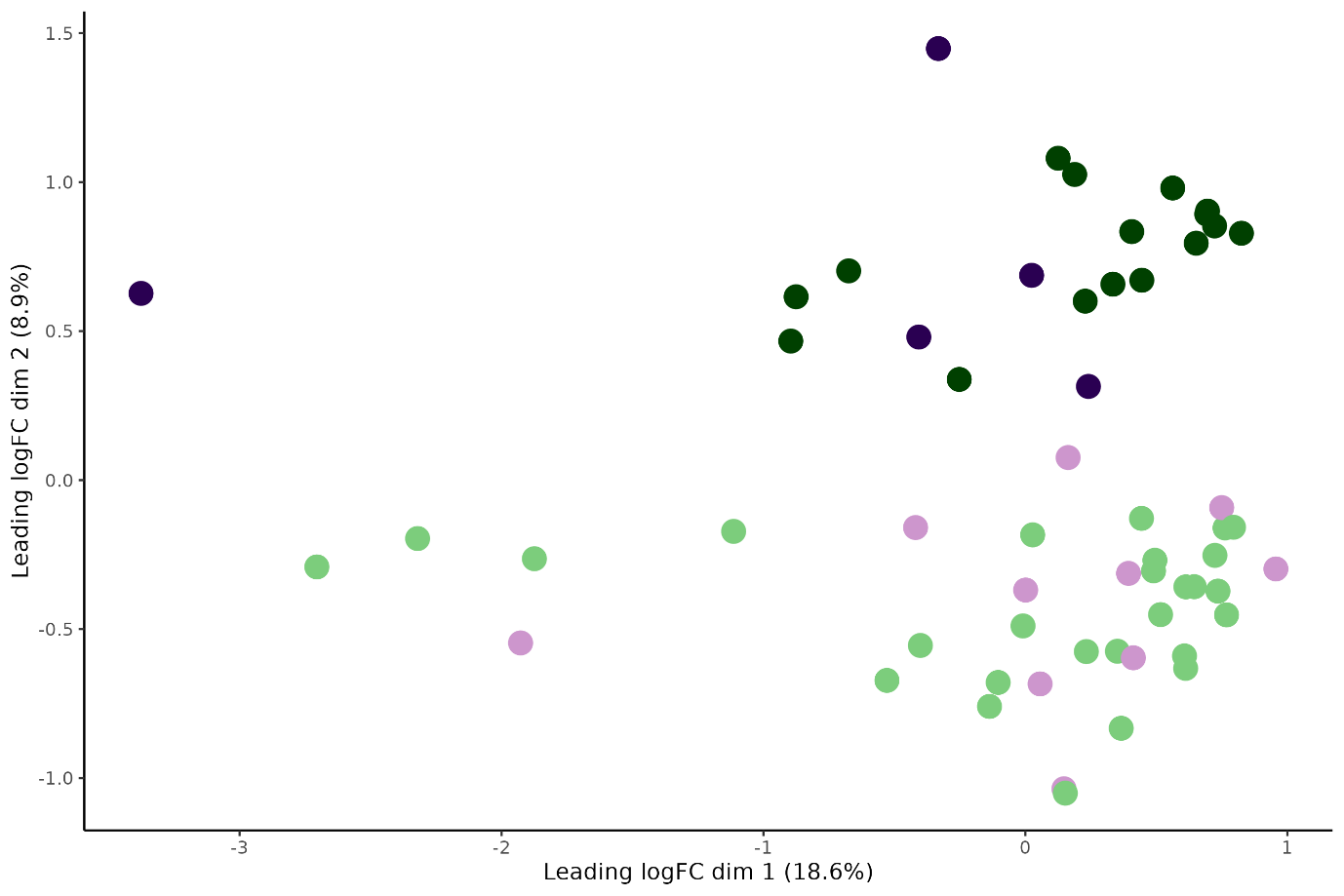
**Figure 1.** *Multidimensional scaling plot of sample data highlights a separation by fetal sex (dimension 2) but not by preeclampsia outcome or maternal demographic characteristics.* Each point represents a sample with distance between points reflects the similarity or dissimilarity of their gene expression profiles. The x- and y-axis show the leading log fold change (average of the largest absolute log fold changes) between samples for dimension 1 and 2 respectively. Male and female fetal sex and outcome visualised as female uncomplicated (light green), female preeclampsia (dark green), male uncomplicated (light purple), male preeclampsia (dark purple).

We evaluated the impact of adjusting for maternal BMI and maternal age on differential expression results using log fold change and adjusted *p*-value estimates including Bland-Altman plots (Figure 2 and 3) and model output correlations (Figure 3 and 4). Across all comparisons, log fold change estimates from the covariate adjusted models were highly consistent with the outcome-only model (mean difference ~0, Spearman’s Rho > 0.97 for all male comparisons and > 0.87 for all female comparisons). Adjustment for maternal age resulted in the tightest agreement, while maternal BMI or combined adjustment led to slightly greater, but still modest, dispersion. These findings indicate minimal confounding by maternal age or maternal BMI, supporting the use of the simpler model to maximise interpretability and statistical power.

**Power Analysis**: To assess the statistical power of our sex-stratified differential expression analyses, we conducted a post-hoc power analysis using the RNASeqPower R package (v1.48.0). The analysis assumed a sequencing depth of 30 million reads, a coefficient of variation of 0.4 (consistent with human placental RNA-seq data), and an alpha of 0.05 after Benjamini-Hochberg correction. For the male comparison (5 preeclamptic vs. 16 uncomplicated placentas), the analysis indicated >80% power to detect a 1.5-fold change in gene expression for genes with average expression >10 counts per million. Power was reduced to approximately 60% for smaller effect sizes (1.2-fold) or lower-expressed genes. For the female comparison (10 preeclamptic vs. 27 uncomplicated placentas), power was >85% for 1.5-fold changes but similarly reduced for subtler effects. These results suggest sufficient power to detect moderate-to-large expression changes in both sexes, though the lower power for smaller effect sizes may contribute to the absence of significant differentially expressed genes in the female cohort. This analysis supports the robustness of the significant findings in male-bearing placentas while highlighting potential limitations in detecting subtler effects in female-bearing placentas.


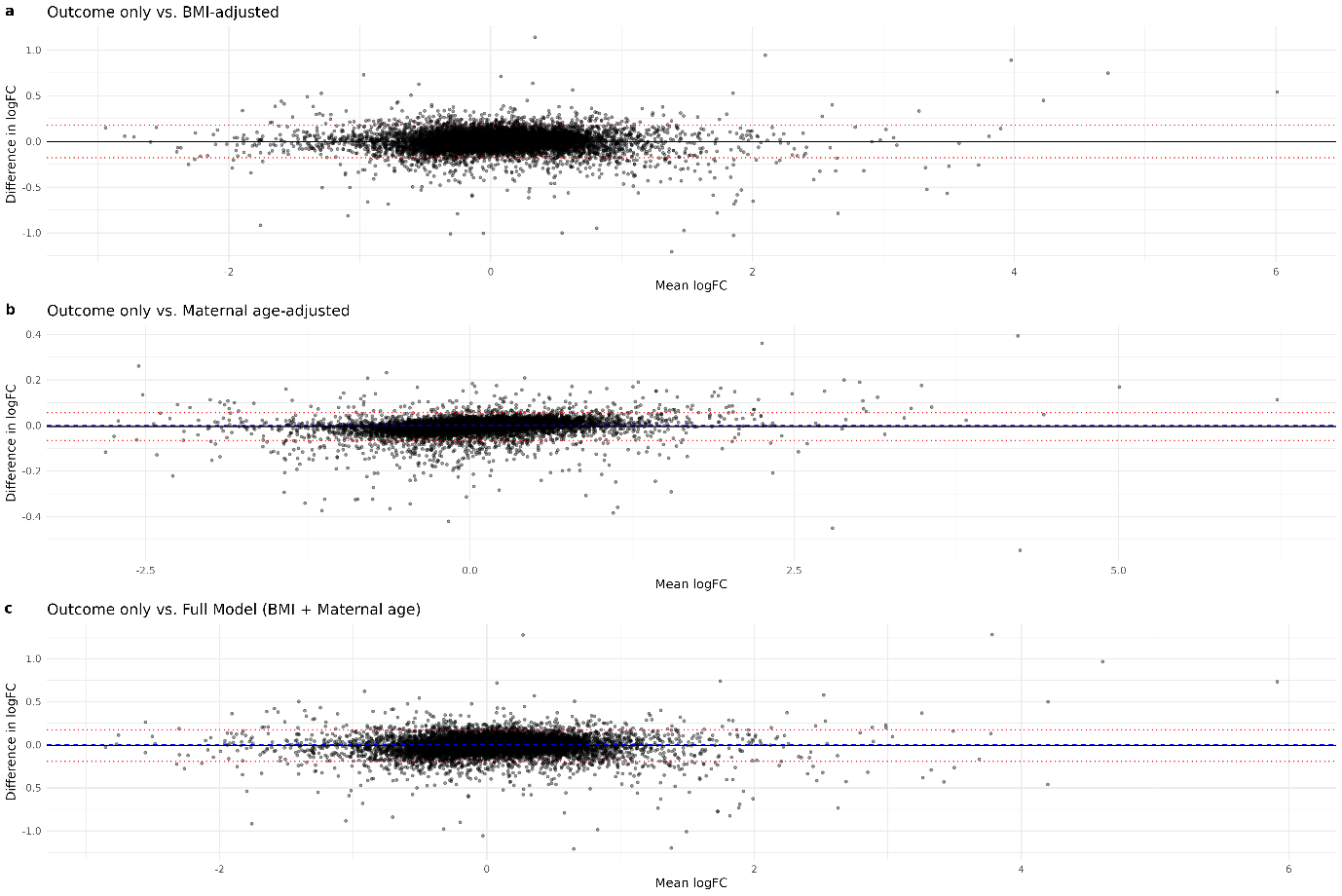


**Figure 2**. *Bland-Altman plots comparing log fold change estimates from models with and without covariate adjustment in male samples.* Each point represents a gene, with the x-axis showing the mean log fold change between the base model (outcome only) and the adjusted model, and the y-axis showing the difference in log fold change. The solid black line denotes the mean difference; red dotted lines indicate 95% limits of agreement and the blue dashed line marks zero. a) Comparison of the outcome only model versus the BMI-adjusted model. A small increase in dispersion is observed, but differences remain centred around zero indicating the maternal BMI adjustment does not introduce systematic bias. b) Comparison with the maternal age-adjusted model reveals tight agreement with minimal spread and no strong bias suggesting maternal age has a negligible effect on log fold change estimates. c) Comparison with the full model (maternal BMI + maternal age) shows a modest increase in variability but overall consistence with the outcome-only model. Together these results support the robustness of the simple model and suggest minimal confounding from maternal age or maternal BMI.


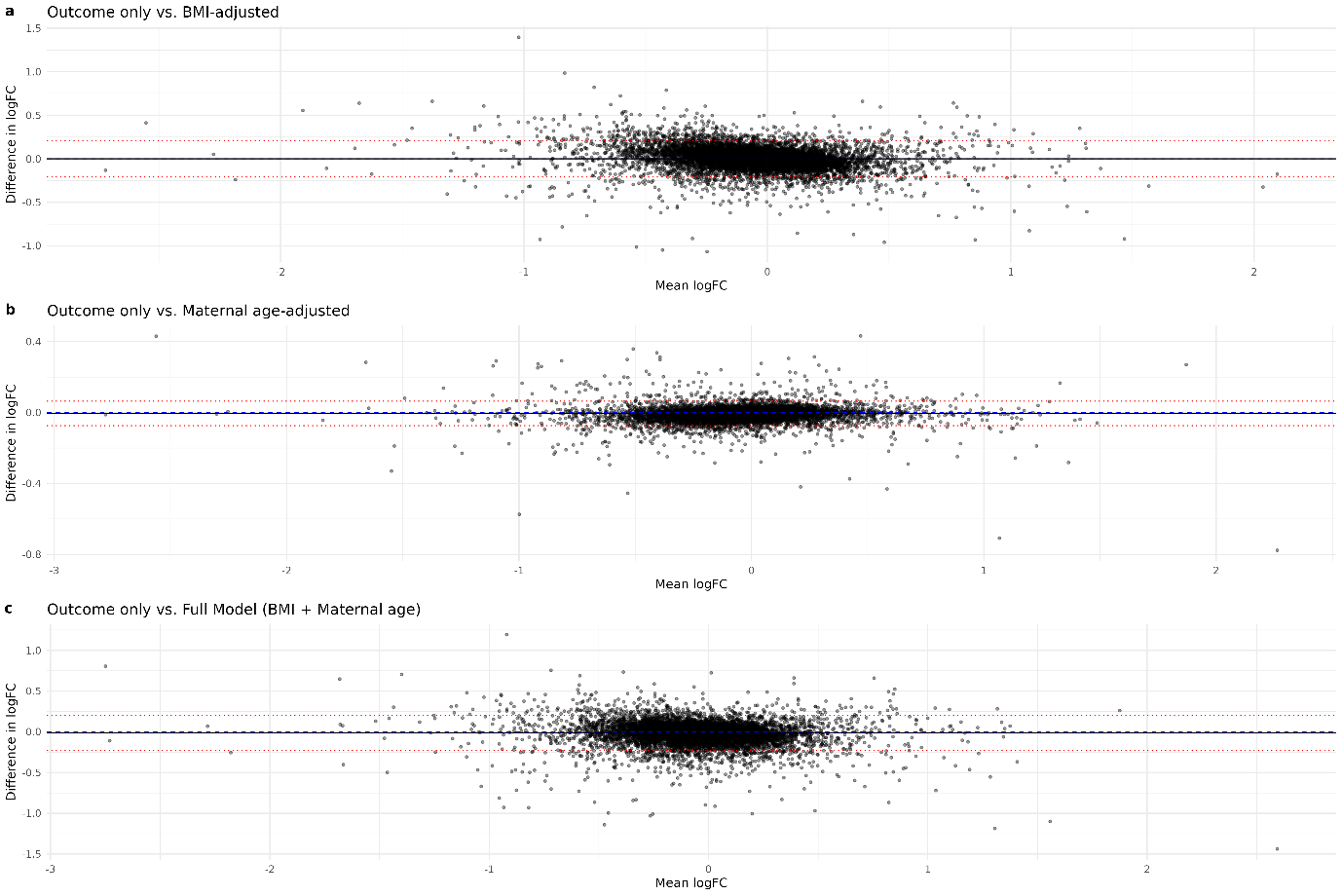


**Figure 3**. *Bland-Altman plots comparing log fold change estimates from models with and without covariate adjustment in female samples*. Each point represents a gene with the x-axis showing the mean log fold change between the base model (outcome only) and the adjusted model, and the y-axis showing the difference in log fold change. The solid black line denotes the mean difference; red dotted lines indicate 95% limits of agreement and the blue dashed line marks zero. a) The comparison with the maternal BMI-adjusted model shows a small increase in variability with most differences remaining close to zero suggesting maternal BMI introduces only minor shifts in gene-level effect sizes. b) Maternal age adjustment yields the most concordant log fold change estimates with tight limits of agreement and minimal dispersion indicating little influence of age on differential expression. c) The full model (maternal BMI + maternal age) exhibits slightly wider limits of agreement but no systematic shift in effect size estimates. This confirms that the combined covariates have limited impact on the core differential expression results.


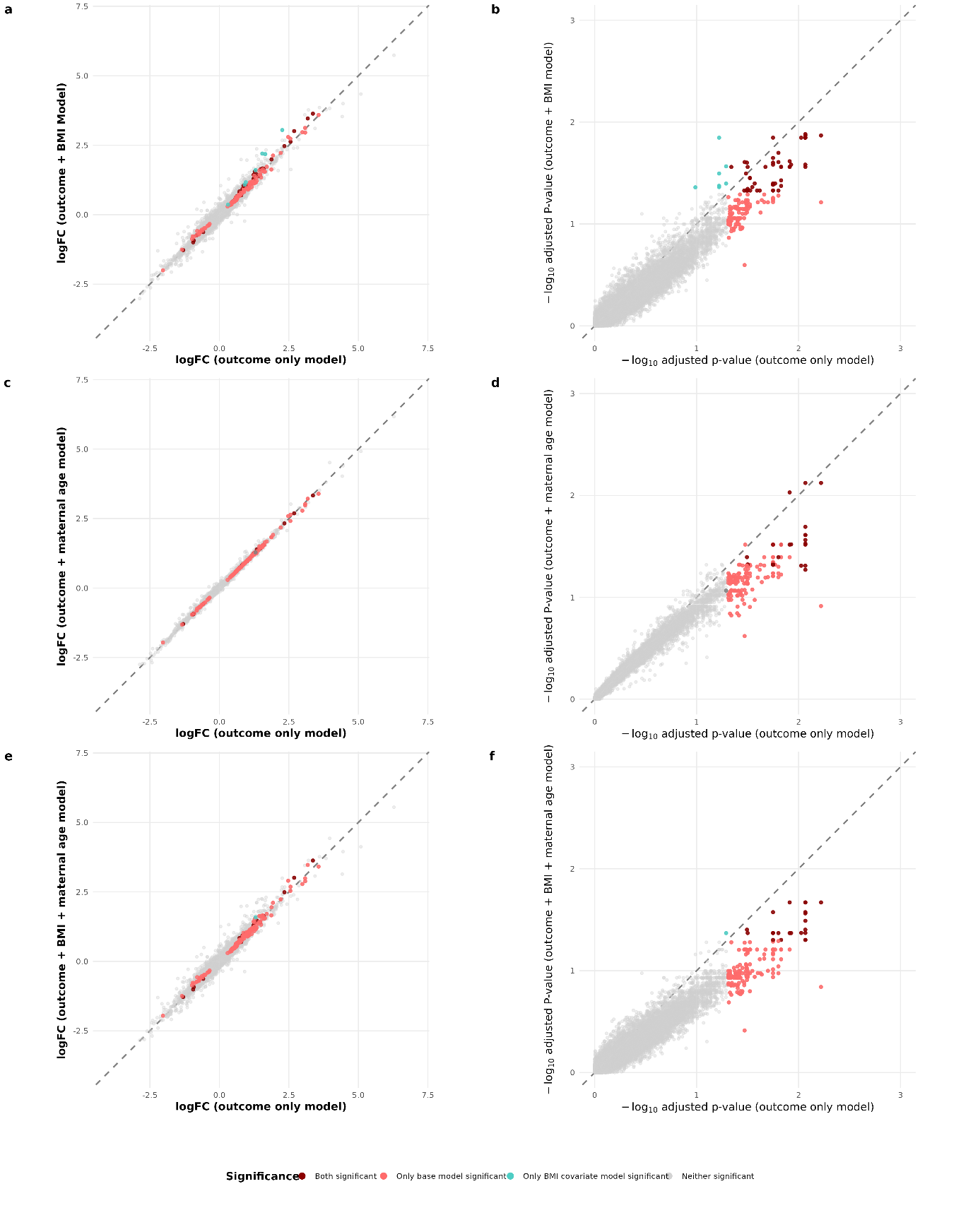


**Figure 4.** *Comparison of log fold change and adjusted p-value estimates from models with and without covariate adjustment in male samples.* Each point represents a gene, with colour indicating significance (red significant in both models, salmon significant in base model only, blue significant in the adjusted model, grey not significant). Plots on the left show the correlation of log fold change estimates between models, while plots on the right compare the corresponding adjusted *p*-values on a -log_10_ scale. Across all comparisons we observed high concordance in log fold change estimates (Spearman’s ρ > 0.97, *p-*value ~0) with the vast majority of genes clustering near the identity line. While adjustment modestly reduced the number of significant genes, consistent with model stringency, overall effect directions and magnitudes were preserved, indicating robust differential expression signals.


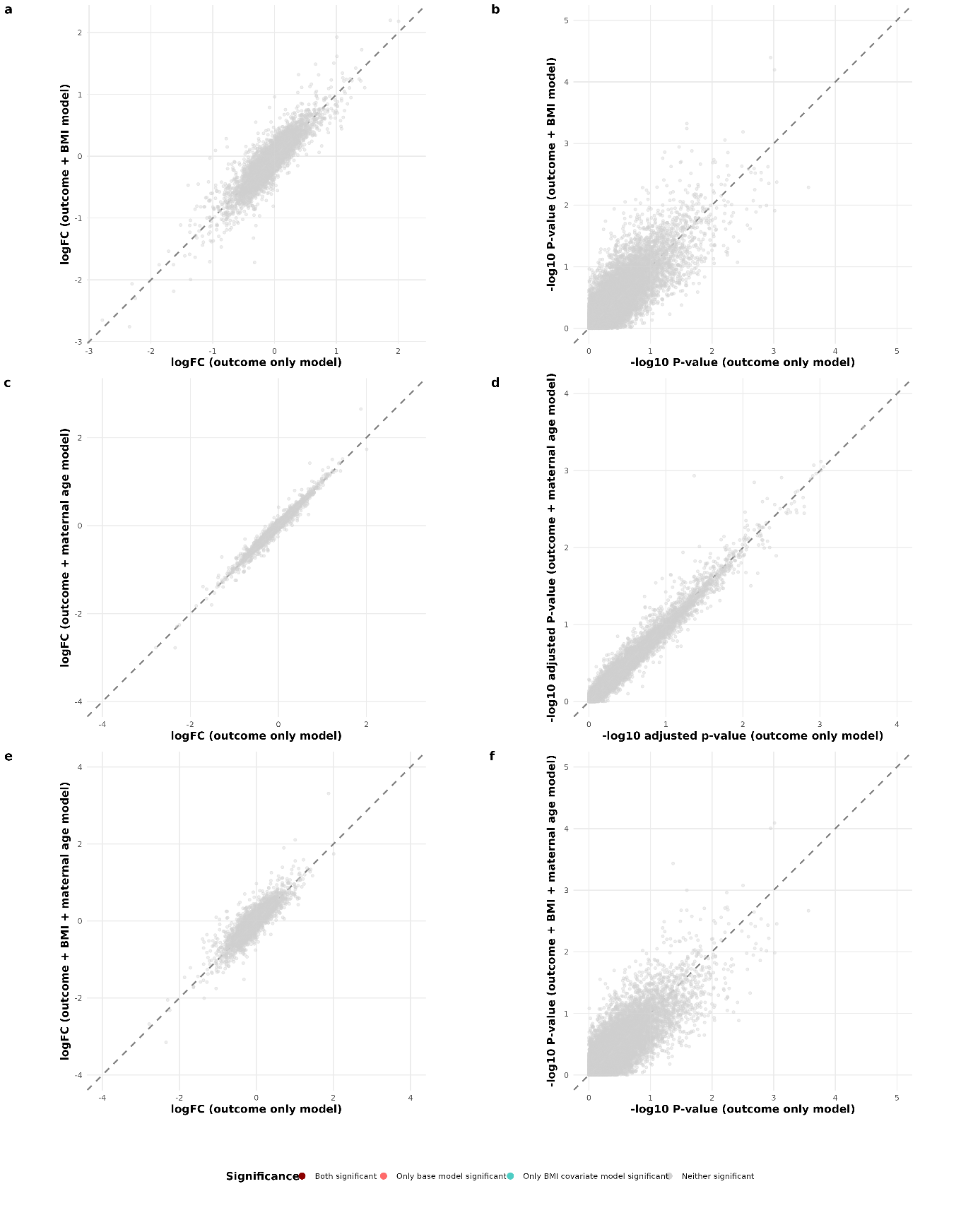


**Figure 5.** *Comparison of log fold change and adjusted p-value estimates from models with and without covariate adjustment in female samples.* Each point represents a gene, with colour indicating significance (red significant in both models, salmon significant in base model only, blue significant in the adjusted model, grey not significant). Plots on the left show the correlation of log fold change estimates between models, while plots on the right compare the corresponding adjusted *p*-values on a -log_10_ scale. Across all comparisons we observed high concordance in log fold change estimates (Spearman’s ρ > 0.87, *p-*value ~0) with increased variation in models containing maternal BMI. However, the vast majority of genes continue to cluster near the identity line. While no statistically significantly differentially expressed genes were identified in the female comparison, the overall effect directions and magnitudes were preserved, indicating robust differential expression signals.
